# Forced treadmill running reduces systemic inflammation yet worsens upper limb discomfort in a rat model of work-related musculoskeletal disorders

**DOI:** 10.1186/s12891-020-3085-z

**Published:** 2020-01-30

**Authors:** Tianqi Tenchi Gao Smith, Ann E. Barr-Gillespie, David M. Klyne, Michelle Y. Harris, Mamta Amin, Ryan W. Paul, Geneva E. Cruz, Huaqing Zhao, Sean Gallagher, Mary F. Barbe

**Affiliations:** 1 Department of Industrial and Systems Engineering, Auburn University, 3323 Shelby Engineering Center, Auburn, AL 36849 USA; 2 Department of Systems Science and Industrial Engineering, SUNY – Binghamton, Vestal, NY USA; 3 College of Health Professions, Pacific University, 190 S.E. 8th Avenue, Suite 230, Hillsboro, OR 97123 USA; 4 Department of Anatomy and Cell Biology, Temple University Medical School, 3500 North Broad Street, Philadelphia, PA 19140 USA; 5 NHMRC Centre of Clinical Research Excellence in Spinal Pain, Injury and Health, School of Health and Rehabilitation Sciences, The University of Queensland, Brisbane, QLD 4072 Australia; 6 Temple University, Philadelphia, PA 19140 USA; 7 Department of Clinical Sciences, Temple University Medical School, 3440 North Broad Street, Philadelphia, PA 19140 USA; 8 Anatomy and Cell Biology, Lewis Katz School of Medicine at Temple University, 3500 North Broad Street, Philadelphia, PA 19140 USA

**Keywords:** Work-related musculoskeletal disorders (WMSDs), Exercise, Tendinopathy, Nerve, Cytokines, Inflammation, Repetitive strain injury

## Abstract

**Background:**

Musculoskeletal disorders can result from prolonged repetitive and/or forceful movements. Performance of an upper extremity high repetition high force task increases serum pro-inflammatory cytokines and upper extremity sensorimotor declines in a rat model of work-related musculoskeletal disorders. Since one of the most efficacious treatments for musculoskeletal pain is exercise, this study investigated the effectiveness of treadmill running in preventing these responses.

**Methods:**

Twenty-nine young adult female Sprague-Dawley rats were used. Nineteen were trained for 5 weeks to pull a lever bar at high force (15 min/day). Thirteen went on to perform a high repetition high force reaching and lever-pulling task for 10 weeks (10-wk HRHF; 2 h/day, 3 days/wk). From this group, five were randomly selected to undergo forced treadmill running exercise (TM) during the last 6 weeks of task performance (10-wk HRHF+TM, 1 h/day, 5 days/wk). Results were compared to 10 control rats and 6 rats that underwent 6 weeks of treadmill running following training only (TR-then-TM). Voluntary task and reflexive sensorimotor behavioral outcomes were assessed. Serum was assayed for inflammatory cytokines and corticosterone, reach limb median nerves for CD68+ macrophages and extraneural thickening, and reach limb flexor digitorum muscles and tendons for pathological changes.

**Results:**

10-wk HRHF rats had higher serum levels of IL-1α, IL-1β and TNFα, than control rats. In the 10-wk HRHF+TM group, IL-1β and TNFα were lower, whereas IL-10 and corticosterone were higher, compared to 10-wk HRHF only rats. Unexpectedly, several voluntary task performance outcomes (grasp force, reach success, and participation) worsened in rats that underwent treadmill running, compared to untreated 10-wk HRHF rats. Examination of forelimb tissues revealed lower cellularity within the flexor digitorum epitendon but higher numbers of CD68+ macrophages within and extraneural fibrosis around median nerves in 10-wk HRHF+TM than 10-wk HRHF rats.

**Conclusions:**

Treadmill running was associated with lower systemic inflammation and moderate tendinosis, yet higher median nerve inflammation/fibrosis and worse task performance and sensorimotor behaviors. Continued loading of the injured tissues in addition to stress-related factors associated with forced running/exercise likely contributed to our findings.

## Background

Musculoskeletal disorders (MSDs) can result from prolonged repetitive and/or forceful movements, and are then referred to as overuse injuries, work-related musculoskeletal disorders, cumulative trauma disorders, or repetitive strain injuries. Work-related MSDs accounted for 34% of all nonfatal occupational injuries and illnesses involving days away from work in the manufacturing sector during 2017 [1]. Work-related MSDs are thought to be a result of one or a combination of risk factors, including physical risk factors (e.g., forceful exertions, repetitive tasks, awkward posture, vibration or chemical exposure), temporal aspects (e.g., work-rest scheduling and work pace), psychosocial risk factors (e.g., low job control, insufficient rest, time pressure, monotonous work, low support from management and coworkers), individual factors (e.g., age, gender, BMI, smoking), among others [2, 3]. The impact of MSDs on the health of workers’ is substantial and broad, and contributes to the incidence and prevalence of chronic pain, anxiety, insomnia, social dysfunction and depression [4, 5].

Treatment of work and repetitive strain injury related MSDs remains challenging because the pathological processes are not fully understood, are complex and involve many biopsychosocial factors [6–8]. It is generally understood that micro-trauma of the tissues heal quickly (i.e., during the subacute phase) unless inflammation is enhanced and/or imbalanced (pro- vs. anti-inflammatory agents), exacerbating tissue damage and fibrosis [9, 10]. Several inflammatory cytokines appear to have critical roles in the development and maintenance of chronic MSDs [11–14]. These small proteins are released by numerous cells that have specific effects on the interactions and communications between immune-related cells, and have critical roles in signaling the movement of cells towards sites of inflammation and trauma [15]. In response to musculotendinous exertion or injury, some cytokines are released directly from the involved skeletal muscle(s) and tendon(s) [16, 17], whereas others are released from neighboring cells such as those in surrounding fascial tissues, and may reach systemically detectable levels [15, 18]. Macrophages that infiltrate injured tissues also produce cytokines through a series of orchestrated pathways [19]. Perhaps not surprisingly, the infiltration of macrophages into nerves is linked to axonal and myelin injury. Human and animal studies in chronic upper extremity work-related MSDs corroborate these findings, showing elevated cytokines systemically and locally in musculotendinous tissues, as well as often collagen matrix changes in and around the injured tissues [8–10, 20–27].

One of the most efficacious treatments for musculoskeletal pain is exercise [28, 29]. Although the pain-relieving mechanisms of exercise remain unclear, evidence points to its capacity to lower systemic inflammation, especially pro-inflammatory cytokines [30, 31]. Positive adaptations have also been observed in and around the exercised tissues, including the down regulation of specific pro-inflammatory cytokines in muscle [32–35] and reduced adipose tissue, which releases a wide-range of cytokines termed adipokines [36]. These local adaptations likely contribute to the lower levels of systemic inflammation observed in physically fit and active individuals. Whether these effects also serve to protect against overuse induced musculoskeletal injuries and pain is unclear.

We have a rat model of work-related MSDs in which rats perform a voluntary high repetition high force (HRHF) upper extremity task of reaching and pulling on a lever bar [37, 38]. We have shown that prolonged performance of this HRHF task induces sensorimotor declines and systemic inflammation [9, 12, 37, 39–42]. This study aimed to test the hypothesis that rats exposed to this model and a concurrent 10 week forced treadmill running regime would have lower systemic inflammation and improved voluntary and reflexive sensorimotor outcomes than rats exposed to the HRHF task alone.

## Methods

### Animal overview

This experiment was approved by the Institutional Animal Care and Use Committee and was compliant with NIH guidelines for the humane care and use of laboratory animals. Twenty-nine female Sprague-Dawley rats were procured at 4–7 months of age from Charles Rivers (King of Prussia, Pennsylvania), housed and handled until reaching young adulthood (3 months of age at the onset of the experiment). Female rats were used exclusively because: 1) higher incidences of work-related musculoskeletal disorders occur in human females than males [43–45], 2) sex is a potential confounder, and 3) results can be compared with those from our previous interventional studies using female rats [12, 41, 42, 46–48]. Animals were housed individually in standard rat cages (ventilated and with hardwood chip rodent bedding) with free access to water in an AAALAC-accredited animal facility with a 12- h light: 12-h dark cycle. Rats were handled at least 3 times per week to reduce investigator-induced stressors, and were provided cage enrichment toys including chew bones. Rats were inspected weekly and postmortem for illnesses and tumors that could contribute to systemic inflammation; none were observed. To reduce the potential for illness-related confounders, additional sentinel rats were examined for presence of illnesses as part of regular veterinary care; none were detected.

All rats included in the study were food-restricted to body weights of no more than 10% less than age-matched free-access-to-food normal controls to encourage involvement in the “food reward-based” lever-pulling task. Normal control rats were used for weight comparisons only, and were not included in the study. All rats in the experiment were weighed twice per week, provided with regular rat chow daily (PicoLab Rodent Diet 5053, Lab Diet, Durham, NC) and food reward pellets (Banana flavored dustless precision pellets; F0024, 45 mg, Bio-Serv, Flemington, NJ) during task performance and allowed to gain weight over the course of the experiment, since they were young adult rats at the onset of the experiment. Food restricted control (FRC) rats that did not perform the task were provided similar amounts of food reward pellets as task rats.

**Fig. 1:**
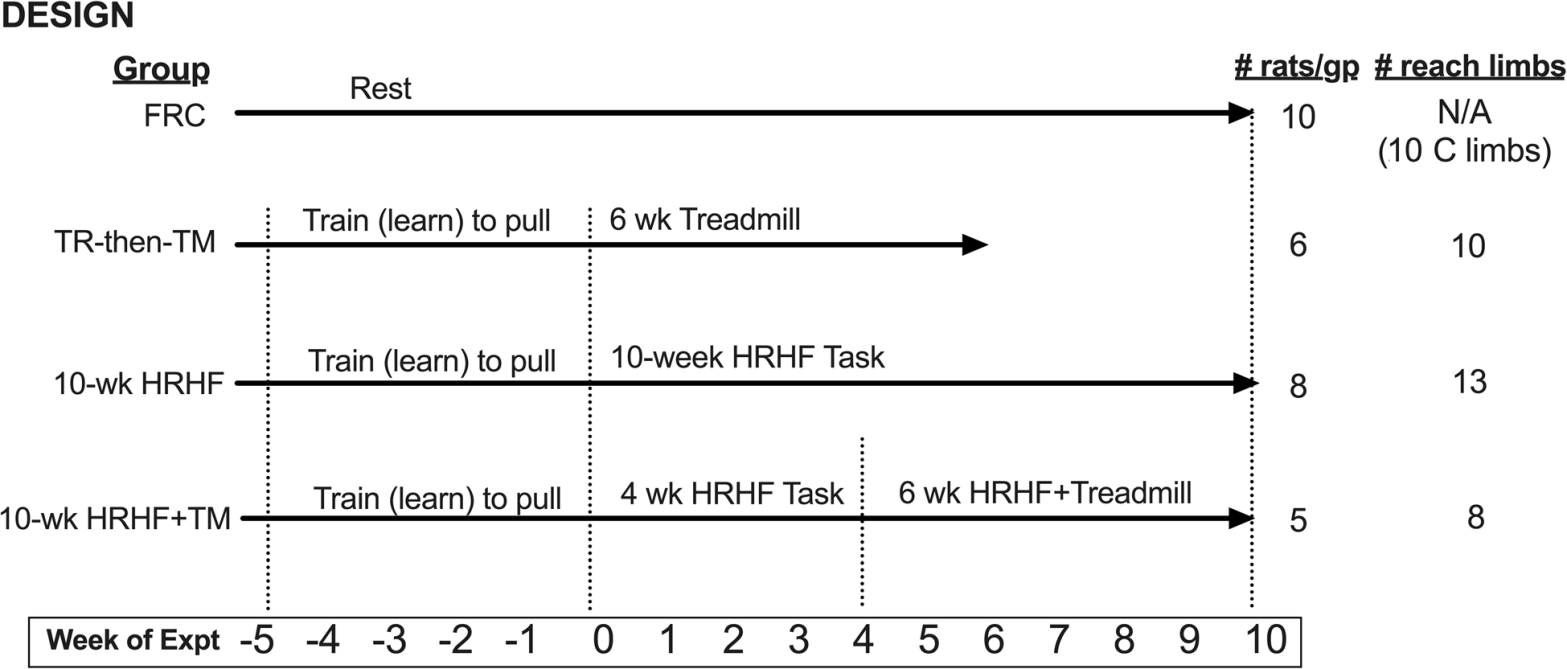
Experimental Design. Twenty-nine young adult female Sprague-Dawley rats were used. Rats were randomly assigned to the groups. There were 10 food restricted only control rats (FRC) that went through no training or task performance. Nineteen additional rats were first trained for 5 weeks to learn pull the lever bar at high force levels, at no specific reach rate. Six of the trained rats did not progress to the reaching and lever pulling task, but instead were engaged only in treadmill exercise running for 6 weeks (TR-then-TM). Eight more of the initially trained rats went on to perform a high repetition high force (HRHF) reaching and lever pulling task for 10 weeks without any intervention (10-wk HRHF). Five more of the trained rats performed the HRHF task for 10 weeks, and while also engaged in flat treadmill exercise running during their last 6 weeks of task performance (10-wk HRHF+TM). A number of rats used both limbs to reach. Therefore, we included data from both reach limbs individually (since task exposures could differ) where appropriate (reflexive grip strength, paw withdrawal thresholds, nerve and muscle macrophage numbers, extraneural fibrosis, and tendon histological assays) for ten TR-then-TM, thirteen 10-wk HRHF rats and eight 10-wk HRHF+TM rats

As shown in Fig. 1, rats were randomly assigned to one of four groups. Nineteen rats were first trained for 5 weeks (15 min/day, 5 days/week) to learn to pull the lever bar at high force levels, at no specific reach rate, as previously described [37]. Eight of these trained rats then performed a high repetition, high force reaching and lever pulling task for 10 weeks without any intervention (termed hereafter as the 10-wk HRHF group; 2 h/day in four 30 min sessions, 3 days/wk), as previously described and depicted [49], and as described further below. A further five trained rats performed the 10-wk HRHF task in addition to forced treadmill running 1 h/day, 5 days/wk. during the last 6 weeks of the HRHF task (termed hereafter as the 10-wk HRHF+TM group), as described further below. The remaining 6 trained rats did not progress to the reaching and lever pulling task, but instead were engaged only in treadmill running for 6 weeks (TR-then-TM group). Four of the six TR-then-TM rats used both limbs to reach, as did five of the ten 10-wk HRHF rats, and three of the five 10-wk HRHF+TM rats. Therefore, we included data from both reach limbs individually (since task exposures could differ) where appropriate (reflexive grip strength, paw withdrawal thresholds, nerve and muscle macrophage numbers, extraneural fibrosis, and tendon histological assays (ten TR-then-TM, thirteen 10-wk HRHF rats and eight 10-wk HRHF+TM rats). Results were compared to 10 food restricted control rats (FRC group; i.e., no training/HRHF task) that were euthanized at matched time points and tissues collected for biochemical and histological assays, using methods described in detail on pages 11–12. FRC rats remained sedentary for the duration of the experiment and underwent handling (3 times/wk), and reflexive sensorimotor testing as per the other animals.

### Behavioral apparatus, training and task regimen

A total of 16 operant rodent chambers were used. Each chamber consisted of a standard open field box placed within a larger sound dampening box (Med Associates, St. Albans, VT) integrated with custom-designed force apparatuses. A metal force lever bar of 15 mm in diameter which task rats were trained to reach and pull on, was placed 2.5 cm outside of each operant chamber wall at the rats’ shoulder height. The lever bar was attached to a miniature tension-compression load cell (LSB200, Futek Advanced Sensor Technology, Irvine, CA) connected with a strain-gauge amplifier (CSG110, Futek). The load cell signal was low pass filtered at 50 Hz and was sampled digitally at 100 Hz by customized Force Lever activity software (ENV-118 M, Product Number SOF-808, Med Associates) that allowed the investigator to select the force level exertion threshold at which the rat received the food reward. Rats were trained to pull the lever at a target force threshold, determined as a percentage of maximum isometric force, for at least 90 ms within a 500 ms auditory cueing window [12]. Training involved learning to reach and pull a lever bar at a force threshold of 60% of the average of all rats’ mean maximum pulling force (MPF, 1.18 Newtons) for 15 min/day, 5 days/wk., for 5 weeks, at no specific reach rate [37]. The HRHF task was a repetitive reaching and lever-pulling task for 10 weeks for a food reward. The specifics of this task were pulling the lever bar at 60% of the rats’ maximum pulling force, a reach rate of 4 reaches/min, for 2 h/day, in 30 min intervals (with 1.5 h break between session), for 3 days/wk. If the lever bar was pulled according to these criteria, a reward light flashed indicating the dispensing of a 45 mg food pellet (Bioserve, NJ) into a trough at floor height [12]. The limb use to reach and grasp the lever bar was tracked for all training and task animals across the course of the experiment.

### Forced treadmill running

Flat running, either immediately after training (TR-then-TM rats) or during the last 6 weeks of the HRHF task (10-wk HRHF+TM rats), was performed on a treadmill (Columbus Instruments) for 1 h/day, 5 days/wk., at a speed ramping up to 23 m/min for 20 min just before start of the dark cycle. Electric shock was not used to avoid stressing the rats. Instead, lab staff prompted the animal to continue running with gentle prodding.

### Voluntary task performance outcomes

HRHF voluntary reaching and lever pulling outcomes were recorded continuously by the Force Lever Program during each task session, for later calculation of reach performance data via an automated script (MatLab; Mathworks, Natick, MA) and then extraction into Excel [12, 42]. Briefly, grasp force was calculated as the average recordable force (expressed as a percentage of maximum pulling force, assayed in Newtons) applied to the force handle for all reaches on a given day. Grasp time was calculated as the average time (in seconds) spent exerting force on the lever bar for all pulls per day. Reach rate was quantified as the average number of reaches per minute (including partial and full pulls on the lever bar) per day. Success rate was expressed as the percent of successful reaches that resulted in a food reward per day out of all recordable reaches. Duration of voluntary task participation per day was calculated as the amount of time (out of 120 min per day) that the rat spent participating in the task rather than sitting in the chamber not pulling. Grasp time and grasp force were calculated using the interval that started when a reach was detected on the lever bar and ended when the force fell below 2.5% of the minimum required force [12]. These voluntary task outcomes were assessed in each of the 10-wk HRHF and 10-wk HRHF+TM groups on the final day of the 10 week task period and encompassed each of the four HRHF task sessions on that day. These data could not be generated for FRC or TR-then-TM rats as they did not perform the task.

### Reflexive grip strength and forepaw/Hindpaw mechanical sensitivity testing

Reflexive grip strength was measured in both forelimbs of all rats using a rat grip strength tester (Stoelting, Wood Dale, IL). The test was repeated 5 times per side. Maximum grip strength of the limbs used to reach was reported for all rats after food restriction, at the end of task week 10 for the 10-wk HRHF and 10-wk HRHF+TM rats, at the end of the 6 week treadmill regimen for the TR-then-TM rats, and at matched time points for FRC rats. The “up-down” von Frey testing method was used for forepaw and hindpaw mechanical sensitivity testing of all rats, bilaterally, as previously described [38] and as we have previously used [12, 46–48]. Monofilaments (North Coast Medical, Morgan Hill, CA) of different diameters were used to elicit a forepaw withdrawal reflex. The force (in grams) of the smallest-sized filament eliciting a withdrawal reflex was recorded as the paw withdrawal threshold (PWT). The person who carried out these assays was an experienced tester and blinded to group assignment.

### Serum and tissue analyses

All animals were deeply anesthetized with a terminal overdose of sodium pentobarbital (120 mg/kg of body weight, i.p., which is in accordance with *AVMA Guidelines for the Euthanasia of Animals*). Depth of anesthesia was assessed and monitored by the pattern and rate of respiration; the absence of muscle tone; and the absence of toe pinch reflex, tail pinch reflex, and eye blink reflex. When the animals no longer showed any reflexive responses, an absence of muscle tone, and breathing had halted, the animals underwent a thoracotomy and blood was then collected from the heart using cardiac puncture with a 23-gauge needle. This took place at 36 h after the final task session was completed in task week 10, in order to avoid possible serum cytokine fluctuations induced by exercise [17, 35, 50, 51]. The blood was stored on ice for ~ 1 h until it clotted before being centrifuged for 20 min at 1000 g at 4^o^ C. Serum (the supernatant) was then collected and stored at -80^o^ C until assayed. Custom rat multiplex ELISA kits from Pierce Searchlight were used to assay serum, in duplicate, for: IL-1α and IL-1β, each pro-inflammatory cytokines; IL-6, a proteic cytokine with both pro-inflammatory and anti-inflammatory properties; IL-10, an anti-inflammatory cytokine; and TNFα, a potent pro-inflammatory cytokine. Corticosterone levels were also assayed in the serum (55-CORMS-E01, Alpco, Salem, NH). Array sensitivity of the serum analytes were: 1.5 pg/ml for IL-1α, 6.2 pg/ml for IL-1β, 6 pg/ml for IL-6, 0.8 pg/ml for IL-10, 3.1 pg/ml for TNFα, and 6.1 ng/ml for corticosterone.

Soleus muscles were then collected (prior to perfusion of now euthanized rats with fixative) for examination of collagen production. Additionally, flexor digitorum muscles were collected from one arm of four of the FRC rats prior to perfusion. These samples were homogenized in phosphate buffered saline containing protease inhibitors, and total protein quantified, as previously described in detail [52]. These samples were prepared for gel electrophoresis by either: 1) boiling and exposing to beta-mercaptoethanol (BME), 2) not boiling the samples before exposing them to BME, 3) or neither boiling nor exposure to BME, in order to detect procollagen, mature collagen or cleavage products, as previously described in detail [52]. All samples were run on a 4–12% Tris-Glycine gel without SDS in the gel, yet with SDS in the sample and loading buffers [52]. After immunoblotting, blots were probed with an antibody against collagen type I (C2456, Sigma-Aldrich, St. Louis, MO). Membranes were stained with Ponceau S prior to antibody probing as a loading control [39].

After anesthesia with a terminal dose of sodium pentobarbital (120 mg/kg of body weight, i.p.) and collection of serum and muscles for western blotting (see above), as described above, animals were perfused intracardially with 4% paraformaldehyde in 0.1 M phosphate buffer using a perfusion pump, before collection of forearm tissues for later histological analyses. The forelimb soft tissue mass (see [53]) was removed from bones *en bloc*, fixed in formalin for 3 days, equilibrated in 10% and then 30% sucrose in 0.1 M phosphate buffer for 2 days each, before being cryosectioned into 14-μm thick longitudinal sections and mounted onto positively charged slides.

Subsets of cryosections containing the median nerve at the level of the wrist were immunostained with an antibody directed against CD68 (a marker of phagocytic macrophages in rats [54–56], Abcam, Massachusetts, United States). After 15 min of 0.5% pepsin antigen retrieval at room temperature, sections were incubated for 20 min in 4% goat serum in phosphate buffered saline (PBS) and then incubated with the anti-CD68 at a 1:250 dilution in PBS at 4^o^ C overnight. The next day, sections on slides were washed 3 × 15 min each, and then incubated with the secondary antibody, AffiniPure F (ab)2 fragment, conjugated to a red fluorescent cyanine dye (Cy3; Jackson ImmunoResearch, West Grove, PA) at a dilution of 1:100 at room temperature for 2 h. When cover-slipping, DAPI was used as a nuclear counterstain. Numbers of CD68+ cells per mm^2^ in the median nerve at the level of the wrist and in the mid-forepaw were quantified, using previously described methods [57] in three to four non-adjacent sections per nerve, and per rat. Nerves were quantified in ten FRC rat forelimbs, and in ten TR-then-TM rats, thirteen 10-wk HRHF rats, and eight 10-wk HRHF+TM reach limbs. This quantification was performed in 3–4 sections/nerve after batch staining by one individual who was blinded to group assignment. Flexor digitorum muscles were similarly examined for presence of immune cells after hematoxylin and eosin staining, and macrophages after immunostaining with anti-CD68 antibody, then a secondary antibody with a horseradish peroxidase (HRP) tag that was detected with diaminobenzidene (DAB) detection methods, followed by eosin counterstaining [53].

Epineurium and extraneural connective tissue thickening was quantified in hematoxylin and eosin stained slides containing branches of the median nerve at wrist-level using a digital camera (R*etiga 4000R QImaging Firewire Camera, Surry, BC Canada*) interfaced with an image analysis system (Life Science, Bioquant Image Analysis Corporation, Nashville, TN). An irregular region of interest (ROI) cursor of 75 μm in size was used to outline the median nerve within the epineurium, and then again at micrometers external to that outline [58]. Then a Videocount Area Array option of the software was utilized (defined as the number of pixels in a field that met a user-defined color threshold of staining) to quantify the number of pixels containing dense pink stained connective tissue within the ROI, relative to the total number of pixels in that region [40]. Three to four sections/nerve were quantified by one individual who was blinded to group assignment. Presence of CD68+ macrophages in epitendons was also examined in sections stained for nerve (see above) to determine if the epitendon cellularity was due to only fibroblast proliferation or also more CD68+ macrophages [53]. The latter was qualitatively examined only.

Subsets of sections of forelimb soft tissues containing flexor digitorum tendon sections were stained with hematoxylin and eosin. Tendons were scored using a semi-quantitative method, the modified Bonar scale, using previously described methods [53]. Briefly, using a scale from 0 to 3, 0 represented a normal histological appearance in the epitendon and endotendon (that is, an elongated cell shape, collagen fibers that were aligned with tenocyte cell shape, and even distribution of cells), while 3 represented advanced pathological changes (e.g., rounded cell shape, wavy fibers, and dense distribution of cells). Tendons were quantified in ten FRC rat forelimbs, and in ten TR-then-TM, thirteen 10-wk HRHF rats, and eight 10-wk HRHF+TM reach limbs. The person who performed the scoring was blinded to group assignment.

### Statistical analyses

An a priori power analysis was performed using data from our prior studies on voluntary task outcomes, grip strength and numbers of macrophages in the median nerve [37, 38, 40]. We chose the most conservative sample size needed to detect differences with an alpha level of 0.05 and 80% power. This a priori power analysis indicated that our estimated sample size needed was 5 per group. Since the observed effect sizes were slightly smaller than the expected values, we performed a retrospective power analysis, which determined that median nerve macrophage results was at 77% power and serum TNFα results was at 79% power. Therefore, we increased the sample size for several assays where appropriate (reflexive grip strength, paw withdrawal thresholds, nerve and muscle macrophage numbers, extraneural fibrosis, and tendon histological assays) by including data from each limb used to reach individually (since task exposures could differ from limb to limb) to 8–13 reach limbs/group (Fig. 1). This increased the power of median nerve macrophage results to 90%.

Next, both Shapiro-Wilk and Kolmogorov-Smirnov tests of normality were performed, and residuals were inspected. Unpaired, two-tailed t-tests were used to compare voluntary reach outcomes at week 10 between the 10-wk HRHF and 10-wk HRHF+TM groups. One-way ANOVAs were used to compare serum cytokines, grip strength, numbers of macrophages in the median nerve, and numbers of immune cells in muscle, using replicate data for the latter two, between all groups. Tukey’s test was used for post hoc analyses; adjusted *p* values are reported. As paw withdrawal thresholds and tendon scores were not normally distributed, Kruskal-Wallis nonparametric tests were used to compare data between groups, and post hoc testing using Dunn’s tests for multiple comparisons; adjusted p values are reported. Pearson’s and Spearman’s rank correlation tests, as appropriate for the data, were used to determine correlations between various outcomes. Significance was set at *p* = 0.05 and results are reported as mean and 95% confidence internals (CI).

## Results

### Serum levels of pro-inflammatory cytokines are lower and Corticosterone higher in the HRHF+treadmill exercise group

**Fig. 2:**
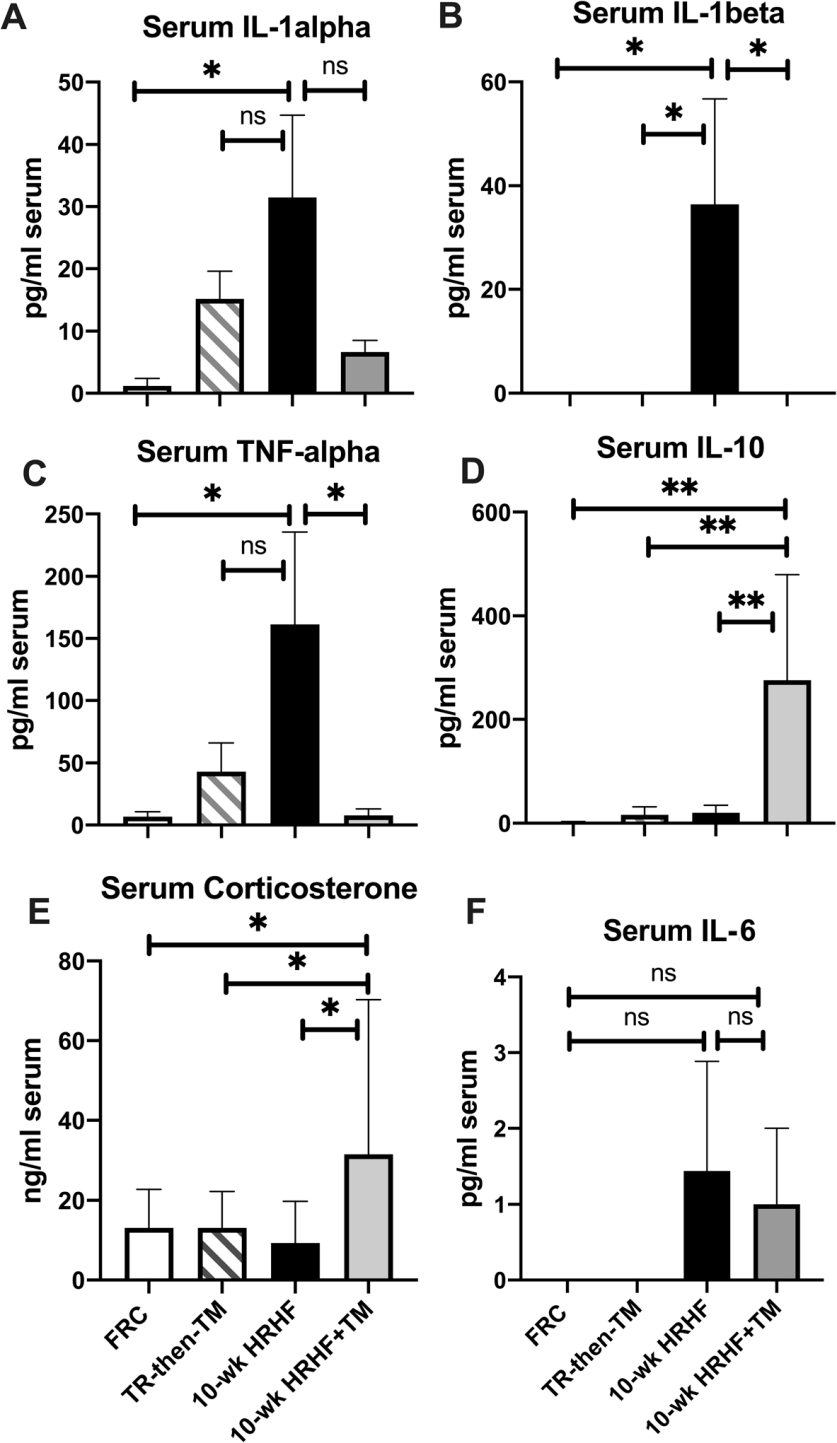
Serum levels of inflammatory cytokines and corticosterone. Serum cytokine levels were assayed using multi-plex ELISA, with data presented as pg of cytokine per ml serum. Serum corticosterone was assayed using single-plex ELISA and data presented as ng/ml serum. **a**-**c** Serum levels of IL-1α, IL-1β and TNFα were highest in the 10-wk HRHF group, compared to FRC animals. IL-1β and TNFα levels were lower in the 10-wk HRHF+TM animals, compared to 10-wk HRHF animals. **d** and **e** IL-10 and corticosterone levels were highest in the 10-wk HRHF+TM animals, compared to the other groups. **f** IL-6 levels did not differ between groups. *: *p* < 0.05, **: *p* < 0.01, and ns = not significant, compared to groups as shown. Mean + 95% CI is shown for: FRC rats (*n* = 10 each analyte), TR-then-TM (*n* = 6), 10-wk HRHF rats (*n* = 8) and 10-wk HRHF+TM rats (*n* = 5)

Several key inflammatory cytokines (IL-1α, IL-1β and TNFα) were higher in 10-wk HRHF animals than FRC rats (Fig. 2a-c). Both groups subjected to treadmill exercise (10-wk HRHF+TM and TR-then-TM) had lower levels of IL-1β, compared to 10-wk HRHF animals (Fig. 2b). TNFα levels were lower in 10-wk HRHF+TM animals, compared to 10-wk HRHF animals (Fig. 2c). In contrast, IL-10 and corticosterone were higher in 10-wk HRHF+TM rats compared to all other groups (Fig. 2d and e). IL-6 did not differ between groups (Fig. 2f), and all analytes did not differ between the TR-then-TM and FRC rats (Fig. 2a-f).

### Voluntary task performance worsens in the HRHF+treadmill exercise group

**Fig. 3:**
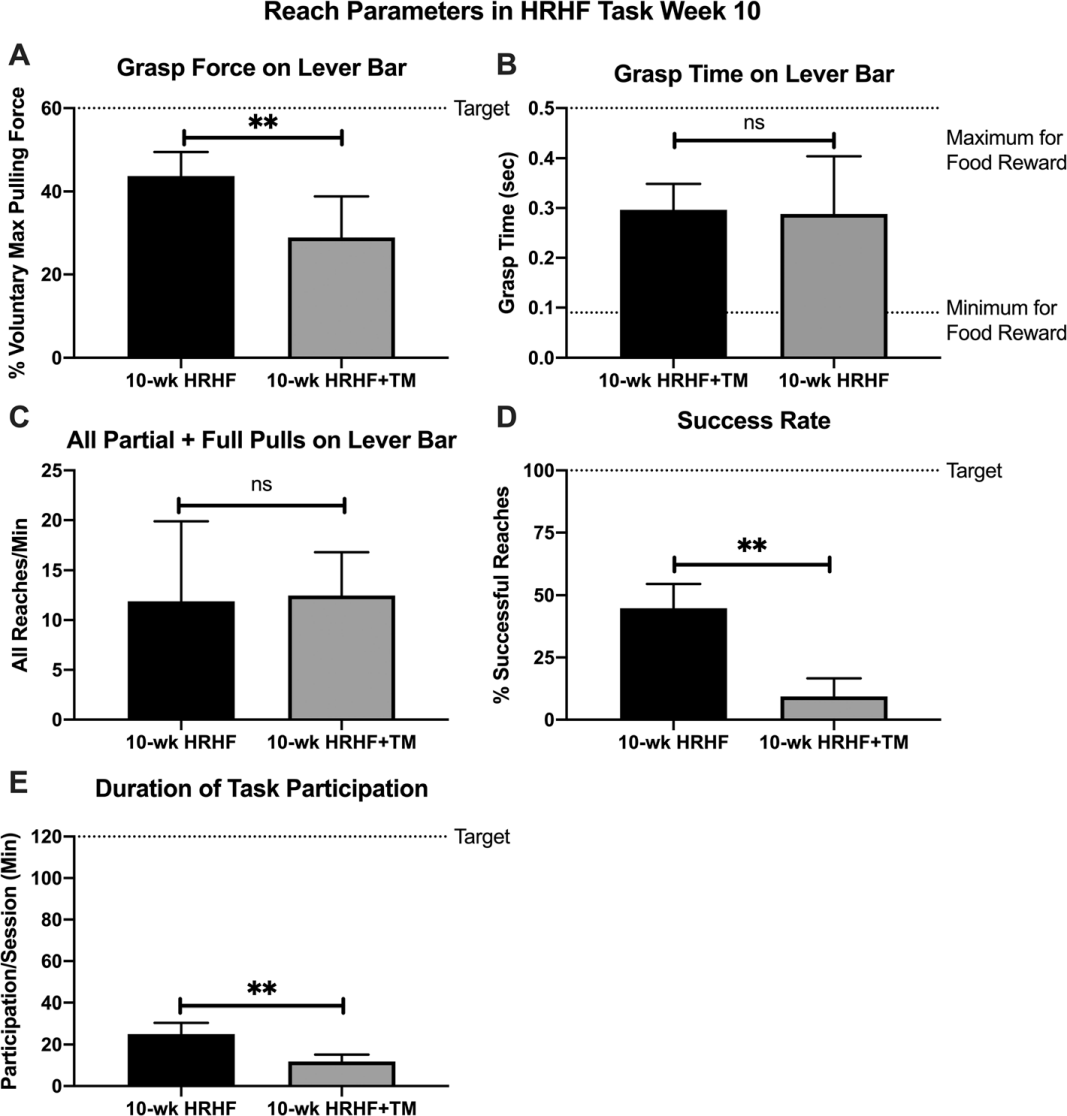
Voluntary Task Performance. **a** Grasp force: percentage of maximum pulling force exerted by pulling on lever bar. **b** Grasp time: time spent grasping and exerting force on the lever bar. **c** Reach rate: number of partial and full pulls on lever bar per minute. **d** Success rate: percentage of successful reaches of all reaches per day. **e** Duration of voluntary task participation: Time spent participating per day, in minutes, with 120 min per day as the target. Grasp force, success rate and duration of task participation were lower in 10-wk HRHF+TM rats, compared to 10 wk. HRHF rats (**:*p* < 0.01 each). There was no significant difference (ns) between the two groups for grasp time and reach rate. Mean + 95% CI is shown for 10-wk HRHF rats (*n* = 8) and 10-wk HRHF+TM rats (*n* = 5)

In task week 10, voluntary grasp force on the lever bar was lower in 10-wk HRHF+TM rats, compared to 10-wk HRHF rats (Fig. 3a). In contrast, grasp time and reaches per minute did not differ between the two task groups (Fig. 3b-c). Success rate was generally poor in both task groups, yet lower still in 10-wk HRHF+TM rats (Fig. 3d), as was the duration of voluntary task performance per day (Fig. 3e). Results point to enhanced discomfort in the HRHF+TM group.

### Heightened forepaw mechanical sensitivity in the HRHF+treadmill exercise group

**Fig. 4:**
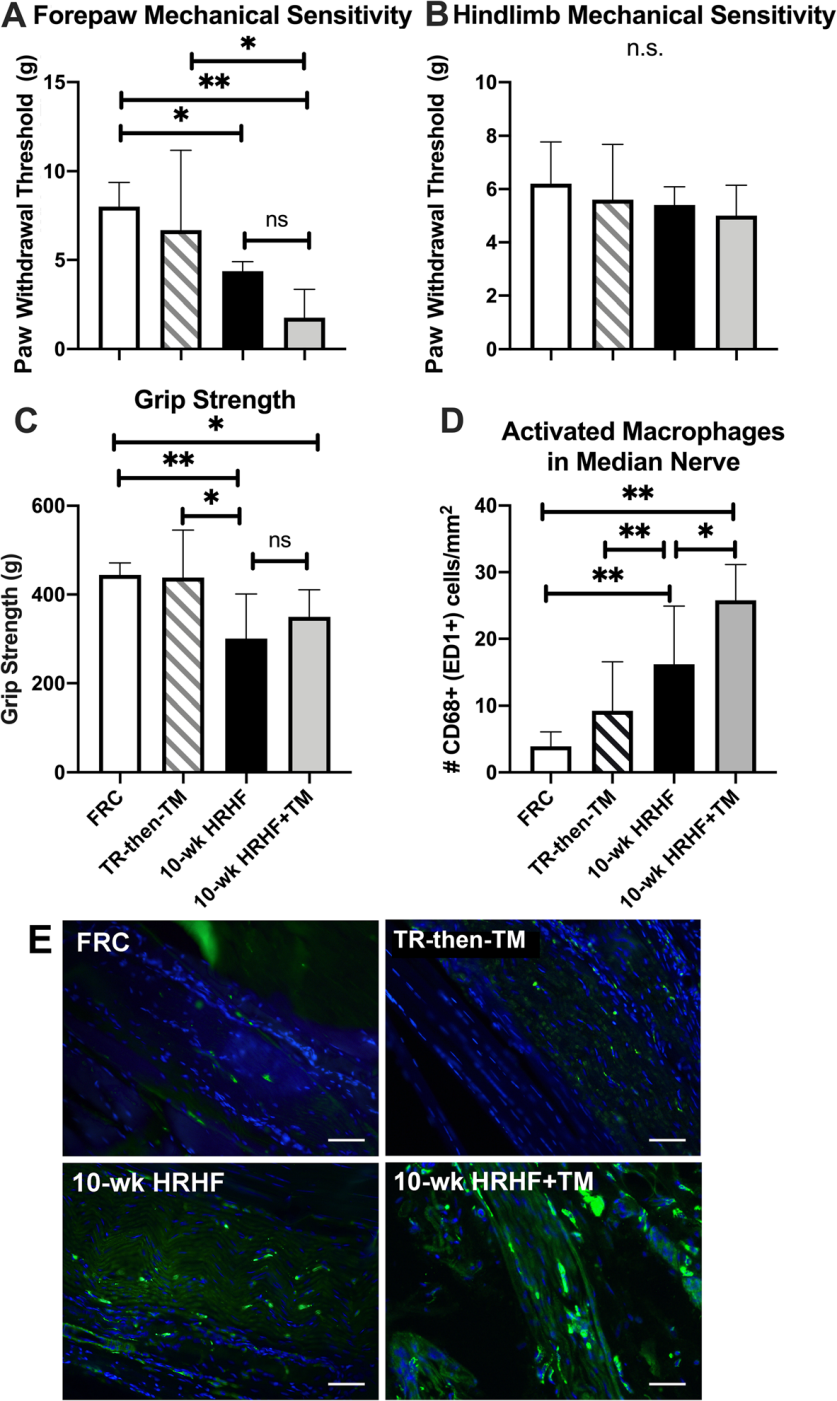
Reflexive sensorimotor declines and median nerve inflammation. **a** Forepaw mechanical sensitivity, expressed as the force (in grams) of the smallest-sized filament eliciting a withdrawal reflex was recorded as the forepaw withdrawal threshold, was lower in 10-wk HRHF and 10-wk HRHF+TM animals, compared to FRC animals. **b** Hindpaw mechanical sensitivity did not differ between groups. **c** Reflexive grip strength was lower in 10-wk HRHF and 10-wk HRHF+TM animals, compared to FRC animals. **d** Quantification of the number of activated macrophages (CD68-immunopositive) in the median nerve at wrist level, showing higher numbers in both HRHF task groups than in FRC animals, and higher numbers in 10-wk HRHF+TM animals than in 10-wk HRHF rats. * and **: p < 0.05 and p < 0.01, compared to groups as shown. Mean + 95% CI is shown for: FRC rats (n = 10), TR-then-TM (n = 10 reach limbs), and 10-wk HRHF rats (*n* = 13 reach limbs) and 10-wk HRHF+TM rats (n = 8 reach limbs). **e** Representative images of CD68+ macrophages (green fluorescence) in median nerves at the level of the wrist, showing similar numbers in FRC and TR-then-TM rats, yet higher numbers in 10-wk HRHF and 10-wk HRHF+TM rats than in FRC rats (with the most in 10-wk HRHF+TM rats). Scale bar = 50 μm. DAPI was used a nuclear counterstain

Both HRHF task groups were more sensitive to mechanical stimuli than the FRC group (i.e., lowered withdrawal thresholds were observed; Fig. 4a). 10-wk HRHF+TM rats were also more sensitive to mechanical stimuli than the TR-then-TM rats (Fig. 4a). No group differences in hindlimb mechanical sensitivity were found (Fig. 4b). Reflexive grip strength was lower in both HRHF task groups, compared to FRC (Fig. 4c). Also, the 10-wk HRHF rats had lower reflexive grip strength than the TR-then-TM rats (Fig. 4c).

### Intraneural inflammatory responses and Extraneural fibrosis was higher in the HRHF+treadmill exercise group

Median nerve inflammatory changes were observed as higher numbers of CD68+ macrophages in median nerve branches at the level of the wrist in both HRHF task groups, compared to the FRC group (Fig. 4d and e). Higher numbers of CD68+ macrophages were quantified within median nerves of 10-wk HRHF+TM rats, compared to 10-wk HRHF rats (Fig. 4d and e). As shown in Fig. 4e, CD68+ macrophages were located within the median nerve (i.e., intraneurally).

**Fig. 5:**
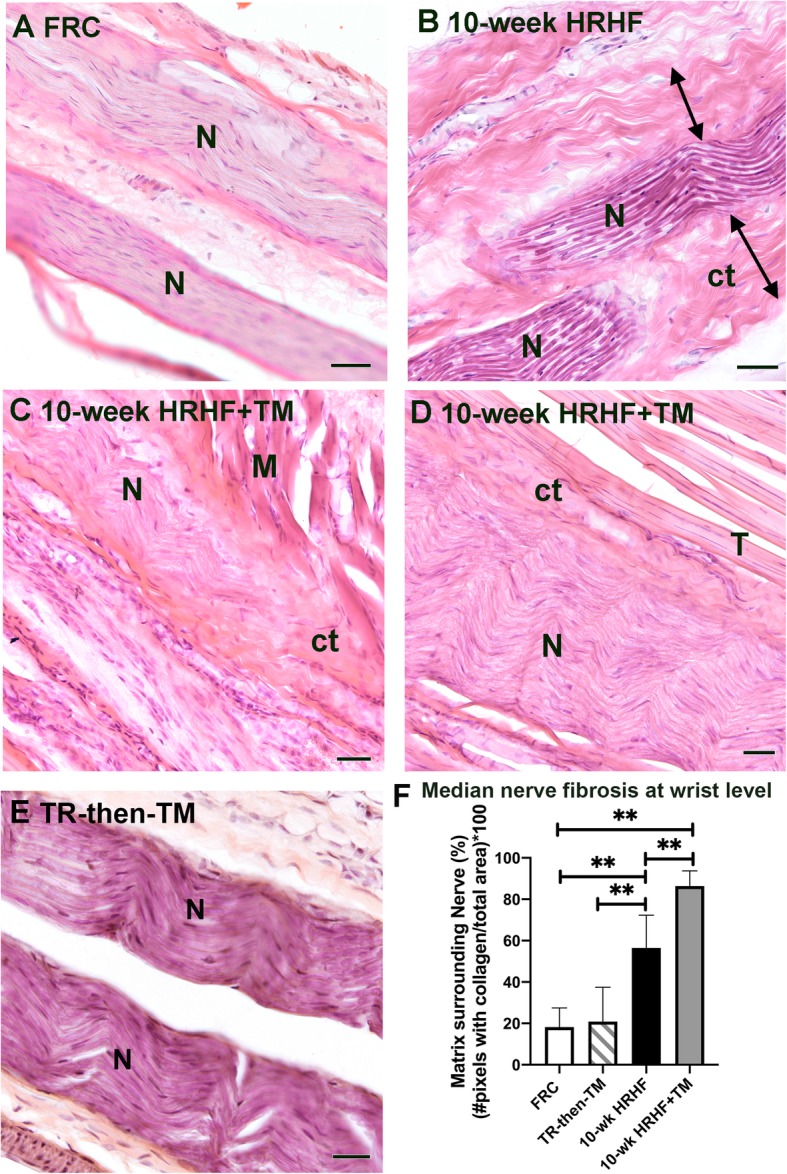
Increased extraneural fibrosis around median nerve of task rats. **a**-**e** Representative images of median nerve branches (N) at the level of the wrist in a FRC rat (**a**), 10-wk HRHF rat (**b**), from two different 10-wk HRHF+TM rats (**c** and **d**), and TR-then-TM rat (**e**). Double-headed arrows in Panel B points out the spread of the extraneural connective tissue into surrounding loose areolar connective tissue in a 10-wk HRHF rat. Panels **c** and **d** show that the expanded epineurium appears connected to muscles (M) and tendons (T) in the 10-wk HRHF+TM rats. Panel E shows that a nerve from a TR-then-TM rat looks histologically similar to a FRC rat. Ct = connective tissue; M = lumbrical muscle; N = nerve; T = tendon. Scale bar = 25 μm. **f** Quantification of extraneural fibrosis at the level of the wrist, within a 50 μm distance from the edge of the nerve/epineurium juncture. **: *p* < 0.01, compared to groups as shown. Mean + 95% CI is shown for: FRC rats (n = 10), TR-then-TM (n = 10 reach limbs), and 10-wk HRHF rats (n = 13 reach limbs) and 10-wk HRHF+TM rats (n = 8 reach limbs)

Regarding the extraneural fibrosis, only a thin layer of epineurium (outer dense connective tissue surrounding nerves) was seen around median nerve branches at wrist level of FRC rats (Fig. 5a). However, this dense connective tissue was thicker around median nerve branches in 10-wk HRHF rats (note double headed arrows in Fig. 5b) and in 10-wk HRHF+TM rats (Fig. 5c and d), and expanded into surrounding typically loose areolar connective tissue, indicative of extraneural fibrosis in each group. Unlike the other groups, in 10-wk HRHF+TM rats, median nerve branches appeared to be connected to lumbrical muscles (Fig. 5c) and tendon slips (Fig. 5d) via this extraneural connective tissue, relative to the other groups. Such extraneural fibrosis or neural tethering was not observed in the TR-then-TM animals (Fig. 5e). Quantification of this thickened extraneural connective tissue confirmed these observations (Fig. 5f).

### Intramuscular CD68+ macrophage numbers were lower, yet fibrosis higher in the HRHF+treadmill exercise group

**Fig. 6:**
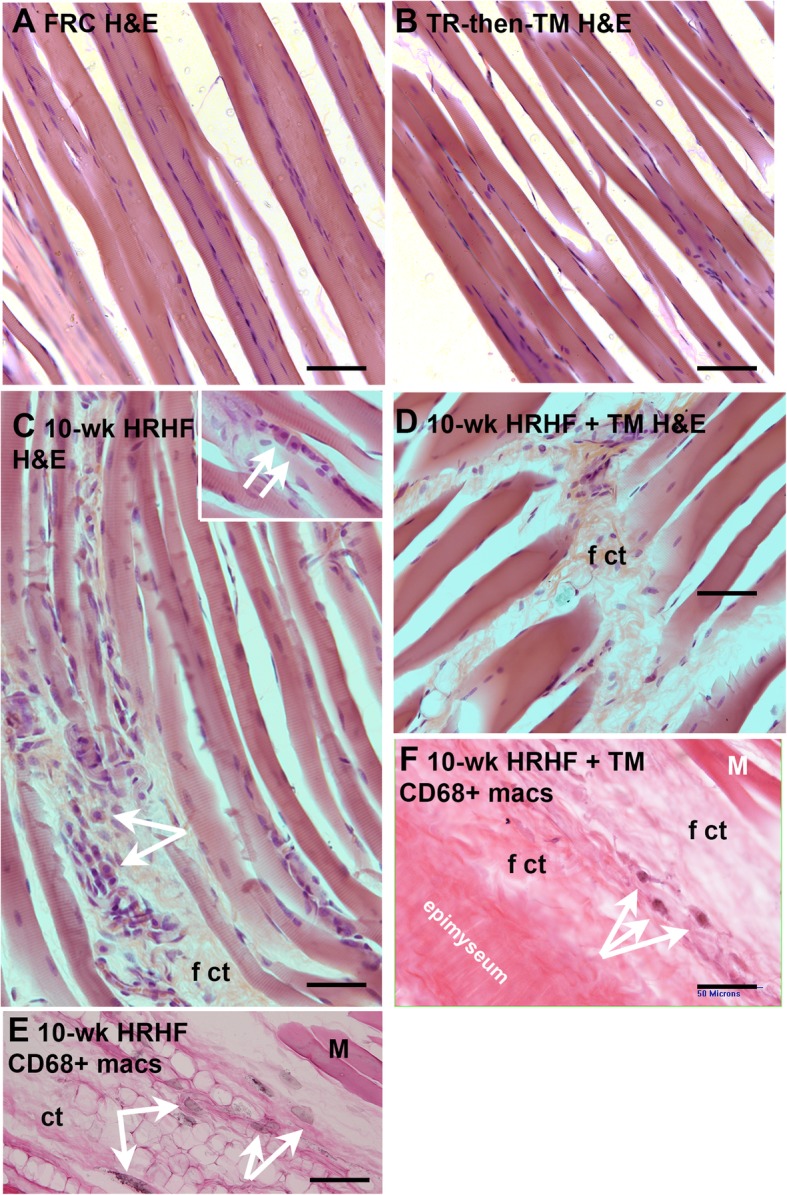
Flexor digitorum muscle pathology. **a** and **b** Representative H&E stained images of flexor digitorum muscles show an absence of enlarged immune cells or intramuscular fibrosis in FRC and TR + TM animals. **c** Representative H&E stained images of a 10-wk HRHF rat muscle showing the presence of immune cells (indicated by arrows). Inset shows additional examples of immune cells (arrows). **d** Representative H&E stained image showing the presence of intramuscular fibrosis (f ct) in a 10-wk HRHF+TM rat muscle. **e** and **f** Arrows indicate examples of CD68+ macrophages (stained black) in muscles of 10-wk HRHF and 10-wk HRHF+TM rats (eosin counterstaining). Scale bar = 50 μm; ct = loose areolar connective tissue; f ct = fibrotic, aka thickened, connective tissue; E = eosin, M = muscle)

Intramuscular regions of the flexor digitorum muscle showed elevated presence of immune cells in 10-wk HRHF rats, relative to FRC and TR-then-TM animals (Fig. 6a-c, examples indicated by arrows in Fig. 6c and its inset). Examination of 10-wk HRHF+TM animals showed presence of intramuscular fibrosis (Fig. 6d and f). Some fibrotic areas were also present in the10-wk HRHF rat muscles, although less than in the 10-wk HRHF+TM animals (compare panels 6C and E, with panels 6D and F). Immunohistochemistry showed elevated presence of CD68+ macrophages in muscles of both 10-wk HRHF and 10-wk HRHF+TM animals (black stained cells indicated by arrows in Fig. 6e and f), although more in the 10-wk + TM HRHF rat muscles. Few to no CD68+ macrophages were observed in FRC or TR-then-TM rat muscles (images not shown). Quantification of these cells revealed higher numbers of immune cells in 10-wk HRHF animals (14.43 ± 8.094, mean ± 95% CI), compared to the other groups: FRC (2.89 ± 5.13), TR-then-TM (4.33 ± 3.68) and 10-wk HRHF+TM (14.43 ± 8.09) animals (*p* < 0.0001 each).

### Distal Epitendon cellularity was higher in the HRHF untreated group

**Fig. 7:**
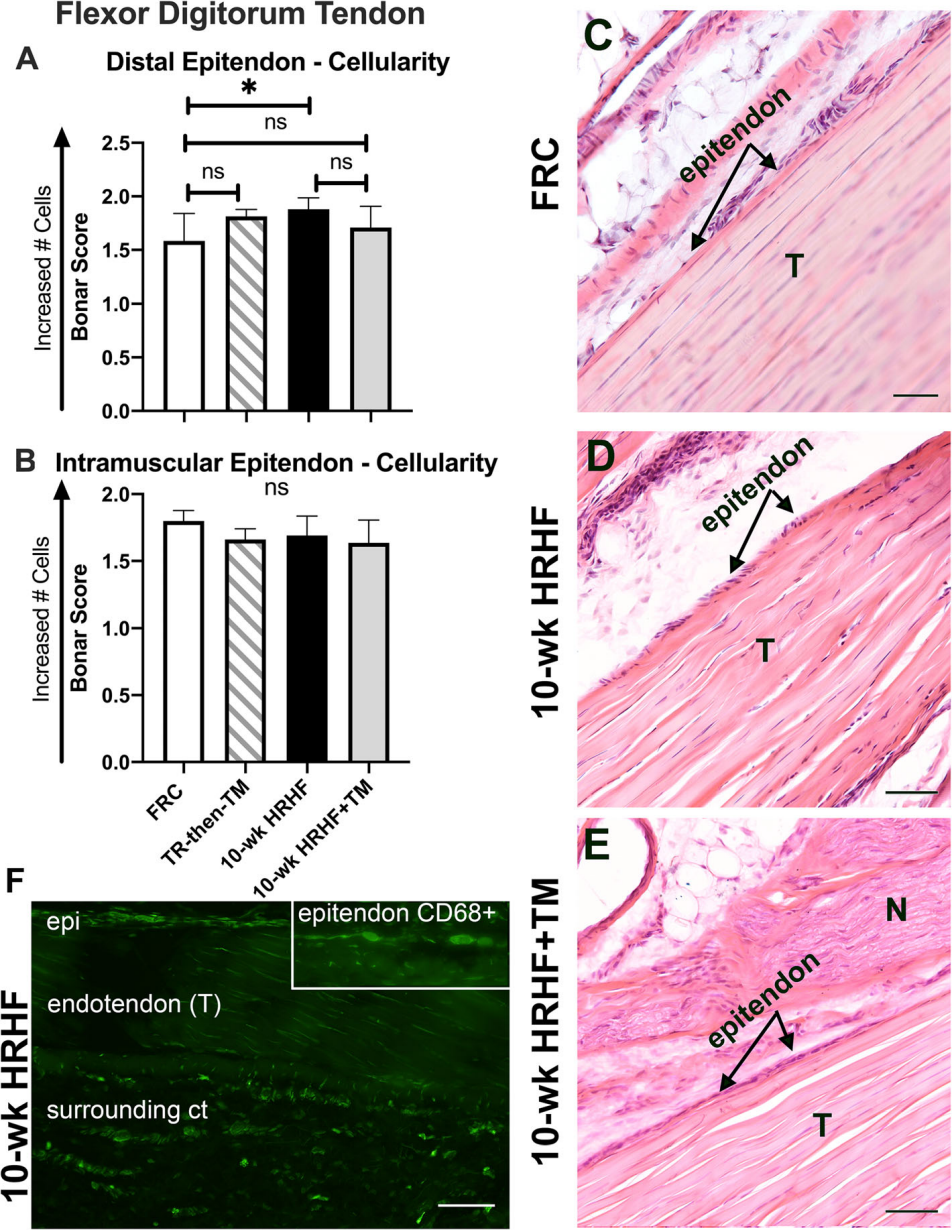
Epitendon cellularity in flexor digitorum tendons scored using a Bonar scoring system. **a** Cellularity score for distal epitendon regions of flexor digitorum tendons. Higher epitendon cellularity was observed in distal regions of flexor digitorum tendons of 10-wk HRHF animals, compared to FRC rats (*: *p* < 0.05). The remaining groups showed no significant (ns) differences in distal epitendon cellularity, compared to FRC rats. **b** Cellularity score for intramuscular epitendons of flexor digitorum tendons, showing no group differences. **a**&**b** For each, the mean + 95% CI is shown for: FRC rats (n = 10), TR-then-TM (n = 10 reach limbs), and 10-wk HRHF rats (n = 13 reach limbs) and 10-wk HRHF+TM rats (n = 8 reach limbs). **c**-**e** Representative images of distal flexor digitorum tendons (T) in a FRC rat (**c**), 10-wk HRHF rat (**d**) and 10-wk HRHF+TM rat (**e**). TR-then-TM rats had similar histological findings as FRC rats (see panel **a**); therefore, a representative image is not included. Some of this higher cellularity in 10-wk HRHF rats’ epitendons was due to an elevated presence of CD68+ macrophages (**f** and inset). Note that there are many CD68+ macrophages in the endotendon and surrounding connective tissues (ct) in the 10-wk HRHF rat image shown. This was not a typical finding in the other groups (images not shown). Scale bar = 50 μm

Cellularity in the distal flexor digitorum epitendon, as quantified using the modified Bonar scale, was greater in 0-wk HRHF rats, compared to FRC rats (*p* < 0.05, Fig. 7a, and panel C vs. D). However, epitendon cellularity within this region was similar between 10-wk HRHF+TM and FRC animals (Fig. 7a and e). Cellularity did not differ between the groups in intramuscular epitendon regions (Fig 7b). There were only moderate, non-significant changes in cell shape, collagen fibril organization or other tendon characteristics between the groups as shown in Fig. 5a-e (images and data not shown). Some of this higher cellularity in 10-wk HRHF rats’ epitendons was due to an elevated presence of CD68+ macrophages (see Fig. 7f and its inset). Note that there are many CD68+ macrophages in the endotendon and surrounding connective tissues in the representative 10-wk HRHF rat tendon image shown (Fig. 7f). This was not a typical finding in the other groups (representative images not shown as they did not differ from FRC rat findings).

### Correlations between serum inflammatory markers, behavior and median nerve findings

**Fig. 8:**
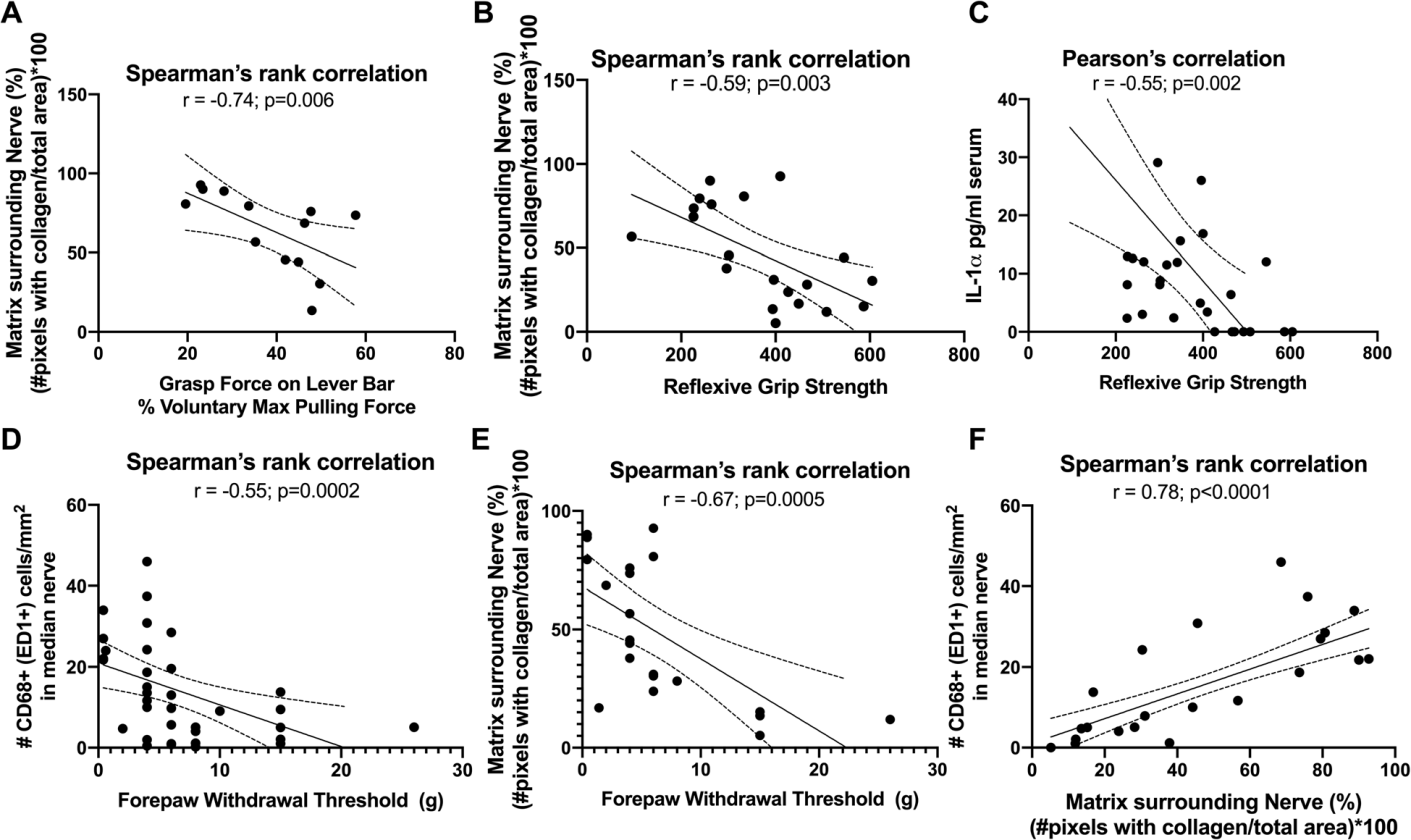
Correlations between serum inflammatory markers, behavior and median nerve findings. **a** and **b** Forelimb voluntary grasp force and reflexive grip strength were moderately and negatively correlated with extraneural fibrosis. **c** Forelimb reflexive grip strength was also moderately and negatively correlated with serum levels of IL-1α. **d** and **e** Forepaw withdrawal thresholds were moderately and negatively correlated with both intraneural inflammation and extraneural fibrosis. **f** A strong positive correlation was observed between extraneural fibrosis and intraneural inflammation

Forelimb voluntary grasp force and reflexive grip strength were moderately and negatively correlated with extraneural fibrosis (r = − 0.74, *p* = 0.006; and r = − 0.59, *p* = 0.003; Fig. 8a and b, respectively). Forelimb reflexive grip strength was also moderately and negatively correlated with serum levels of IL-1α (r = − 0.55, *p* = 0.002, Fig. 8c). However, forepaw withdrawal thresholds were only weakly and negatively correlated with serum levels of IL-1α (r = − 0.42, *p* = 0.02; figure not shown). In contrast, forepaw withdrawal thresholds were moderately and negatively correlated with both intraneural inflammation (r = − 0.55, *p* = 0.0002, Fig. 8d) and extraneural fibrosis (r = − 0.67, *p* = 0.0005, Fig. 8e). A strong positive correlation was observed between extraneural fibrosis and intraneural inflammation (r = 0.76, *p* < 0.0001, Fig. 8f).

**Fig. 9:**
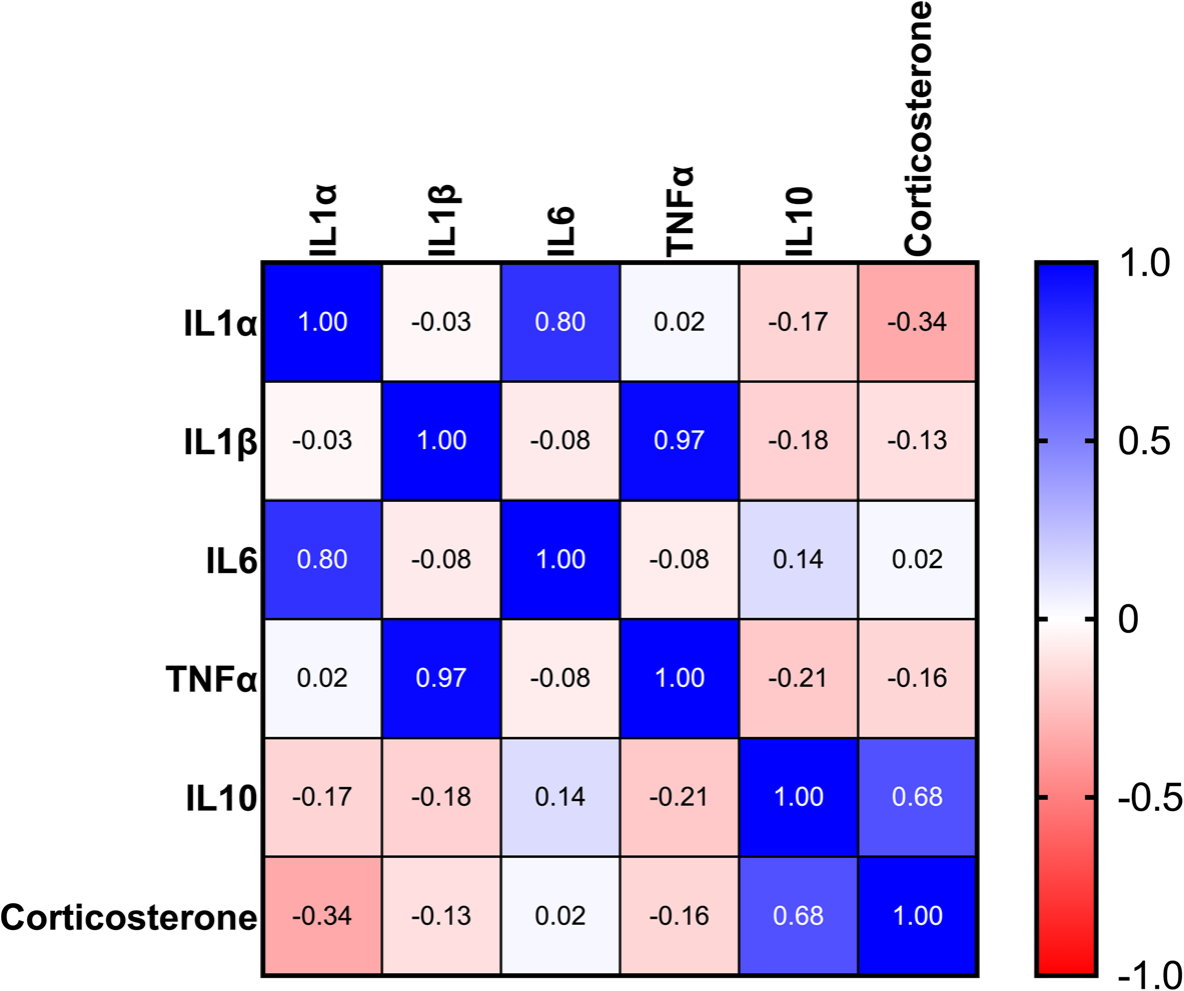
Heat map of correlations between the various serum inflammatory markers and corticosterone assayed. As shown on key on the right, blue indicates the strongest positive correlations, while red indicates the strongest negative correlations. We observed strong positive associations between IL-1α and IL-6 (r = 0.80), and IL-1β and TNFα (r = 0.97), as well as a moderate positive association between IL-10 and corticosterone (r = 0.68). The weak negative correlation observed between IL-1α and corticosterone (r = − 0.34) was not significant (*p* = 0.26)

Several correlations were also observed between various serum cytokines and corticosterone levels (Fig. 9). Strong positive associations were observed between IL-1α and IL-6 (r = 0.80, *p* = 0.00002), and IL-1β and TNFα (r = 0.97, *p* = 0.000001), as well as a moderate positive association between IL-10 and corticosterone (r = 0.68, *p* = 0.01). Although IL-1α and corticosterone tended to weakly correlate (r = − 0.34), the relationship was not significant (*p* = 0.26).

### Collagen type I production in Hindlimb soleus muscles showed low Procollagen synthesis

Since alterations in collagen synthesis is a known physiological adaptation that occurs in skeletal muscle in response to mechanical loading [59], we collected and examined hindlimb soleus muscles. Because only one third of soleus muscles of 10-wk HRHF+TM rats showed more procollagen type I (~ 250 kDa) or mature collagen (~ 130 kDa), relative to FRC hindlimb soleus muscles, the physiological effects of treadmill running on the hindlimb muscles appeared minimal (Additional file 1: Figure S1A). No changes in cleaved collagen were observed (~ 80 kDa and ~ 50 kDa bands; Additional file 1: Figure S1A). Also, examination of hindlimb soleus muscles in 10-wk HRHF+TM rats showed no differences in pro- or mature collagen type I production, relative to FRC flexor digitorum muscles that were not exposed to treadmill running (Additional file 1: Figure S1B).

## Discussion

Consistent with our original hypothesis that treadmill running should be an efficacious treatment in this upper extremity work-related musculoskeletal disorder model [18, 60–62], the pro-inflammatory cytokines IL-1β and TNFα levels were lower and the anti-inflammatory cytokine IL-10 was higher in serum of HRHF+treadmill animals than in the untreated HRHF animals. Further, epitendon cellularity in distal flexor digitorum tendons and immune cell numbers in flexor digitorum muscles were lower in HRHF+treadmill animals than in the untreated HRHF animals. However, inconsistent with our original hypothesis, the combination of HRHF and treadmill running resulted in lower voluntary grasp force, reach success, task participation, reflexive grip strength and forepaw withdrawal thresholds, compared to untreated HRHF animals. In addition, more CD68+ macrophages and extraneural fibrosis were observed within and around median nerves, respectively, in HRHF+treadmill animals. Corticosterone levels were also elevated in the running versus the non-running HRHF groups. To summarize, treadmill running was associated with lower systemic inflammatory cytokines, muscle immune cells and distal epitendon cellularity, but higher corticosterone, worse task performance and sensorimotor behaviors, and median nerve pathology in rats that were simultaneously performing a HRHF task, compared to untreated HRHF rats.

As shown previously, the capacity for rats to perform the HRHF task diminishes over time [9, 37, 38]. However, we have had prior success in reducing performance loss with several interventions, including: 1) daily ibuprofen treatment of rats performing the same HRHF task for 12 weeks (although those improvements were not sustained for the full 6 weeks of treatment) [42]; 2) ergonomic task reduction in which rats were moved from the HRHF task after week 4 to a low repetition, low force task that they continued to perform for another 6 weeks [12]; and 3) modeled manual therapy provided 5 days/week simultaneously with performance of the HRHF task for 3 or 12 weeks [40, 63]. That our treadmill running regimen was associated with worse task performance suggests that it enhanced the negative effects of this upper extremity HRHF task. The latter idea is supported by the enhanced intraneural inflammation in the form of CD68+ cells and nerve fibrosis that was higher in the HRHF+treadmill group than in the untreated HRHF group.

Presence of phagocytic macrophages within nerves is known to be a sign of nerve damage in rats and humans, often as a result of chronic nerve compression [64–68]. Neuritis and nerve injury are associated with increased ectopic nerve firing, heightened nerve sensitivity, and increasing discomfort [58, 63, 69]. The moderate to strong correlations between CD68+ cells, extraneural fibrosis and lower paw withdrawal (i.e., thresholds heightened forepaw mechanical sensitivity) are also consistent with these prior studies, and with studies examining human subjects that have positive clinical signs of carpal tunnel syndrome, including increasing pain and tingling [70]. Since indices of nerve inflammation (CD68+ cells in the median nerve), extraneural fibrosis, forepaw mechanical sensitivity were evident in HRHF rats, and more so in HRHF+treadmill rats (relative to controls), we suggest that the treadmill running enhanced the negative effects of this upper extremity HRHF task.

That systemic inflammation was lower but indices of performance and local tissue pathology were worse in the treadmill group presents an interesting paradox. Reductions in systemic inflammation are often associated with reduced inflammation at the site of injury, decreased pain/discomfort and improved outcomes in numerous musculoskeletal conditions in humans [71, 72] and in this rat model previously [41, 47]. Median nerve cytokine changes released from activated macrophages at the wrist are not likely to be at high enough levels to be systemically detected in rats (the nerve is only 1–1.5 mm in diameter) [73]. Levels of systemic cytokines are more likely to match muscle and bone inflammatory responses [37, 74] (the latter tissue not examined as part of this study). Aerobic exercise is a potent anti-inflammatory (locally and systemically) [31, 62, 75], reduces adipose tissue within and around the exercised tissues [72, 76], enhances muscle fiber formation [77, 78] and promotes axonal regeneration after peripheral nerve injury [79]. In our case, we suspect that the running task had an additive “repetitive” loading effect on the already strained tissues exposed to the HRHF task and contributed to the injurious cycle. This is supported by the lack of significant findings in the TR-then-TM rats (rats that underwent the initial training and then were engaged only in the treadmill running regime while otherwise resting). Encroachment of macrophages and enhanced fibrosis in the affected tissues in HRHF rats that performed treadmill running also support this theory. The forced nature of the exercise may in part explain these findings. There is increasing evidence that the intensity, frequency and/or duration of exercise beyond a certain threshold can increase pain, and that this threshold is determined by various factors such as fitness level and state of the injury, tissues or pain [80]. Exercise beyond this threshold can promote various pain-enhancing changes [80, 81], even in the central nervous system (e.g., increased activation of NMDA receptors in pain modulating areas) [82]. Stress-related problems including increased pain have also been associated with forced exercise [83, 84]. Elevated levels of the stress hormone corticosterone (the cortisol equivalent in humans) in the HRHF treadmill group suggest that stress may have also contributed to negative outcomes. On the other hand, the anti-inflammatory properties of corticosterone [81, 85] may in part explain the low levels of systemic inflammation in this group.

Regarding the low levels of IL-6 in the serum. Many investigators have shown increased circulating levels of IL-6 acutely after exercise in humans and animal models [17, 35, 50, 51]. Yet, IL-6 was not significantly upregulated in the HRHF-untreated or HRHF+TM rats, compared to the control rats. This finding matches those in prior studies using this model, that found no increase in IL-6 in young adult rats engaged in repetitive tasks [74, 86]. This may be because we typically wait 18–36 h after the last task session (and 36 h in this study after the last task and treadmill running session) to collect blood samples in order to avoid muscle activity induced elevations in inflammatory cytokines (which apparently we did, at least for IL-6). Prolonged exercise training is associated with reduced basal IL-6 production [87], so perhaps the 5 weeks of training followed by prolonged repetitive task performance period of 10 weeks also contributed to the low IL-6 serum levels.

Some limitations of this study need to be considered. First, only female rats were included. As the upper extremity work-related musculoskeletal disorder model setup (e.g., force transducer sensitivity) was tailored to the pulling strength of female rats, inclusion of males would have reduced data quality and made the interpretation of findings more difficult, as well as added sex as a potential confounder. That said, although human females have a higher incidence of work-related musculoskeletal disorders than males [43–45, 88], human males also develop these disorders [89–91]. Future studies using this model are encouraged to include male rats. Second, treadmill running turned out to be a counterproductive exercise choice because it directly and repetitively loaded tissues of the limbs that were undergoing task-induced overuse injuries. Other exercise methods that might have avoided or generated less direct loading includes voluntary wheel running and swimming, although swimming is considered highly stressful to rodents [92]. Third, the inclusion of different levels of exercise intensity (i.e., speed, duration and frequency) would have allowed for exploring intensities that produce analgesia and optimal outcomes. The positive or negative effects of voluntary running wheel exercise are currently being examined in this rat model.

## Conclusions

Other than lower systemic inflammation, treadmill running was associated with worse tissue, sensorimotor and performance outcomes in animals that performed the HRHF task. These findings refute the assumption that adaptations to exercise are always positive, and that the intensity, type and potential for psychological stress should be considered when developing exercise interventions aimed at preventing or reducing injury/pain. Whether voluntary (e.g., free access to a wheel) over forced running improves outcomes in animal and human models of work-related musculoskeletal disorders are an interesting area of future research.

## Supplementary information

**Additional file 1: Figure S1.** Western blots of muscles probed for Collagen type I. Expected location of procollagen I, mature collagen type I, and two cleaved collagen bands are indicated. No significant changes in collagen synthesis or cleavage were observed. A) Left panel is a representative Western blot of 10-wk HRHF+TM rats’ soleus muscles and a FRC rat’s soleus muscle, probed with an antibody against collagen type I (Sigma C2456). Lane 1 = Marker; Lanes 2–4 are homogenates of soleus muscles from 3 different 10-wk HRHF+TM rats. Lane 5 is a homogenate of a soleus muscle from a FRC rat. Samples were not boiled, but exposed to BME before running on a 4–12% Tris-Glycine gel without SDS in the gel, yet with SDS in the sample and loading buffers. The right image is the same membrane stained with Ponceau S prior to antibody probing, used as a loading control. B) Left panel is a representative Western blot of a HRHF+TM rat’s soleus muscles compared to a FRC rat’s flexor digitorum muscle, after probing with the same antibody as in panel A (Sigma C2456 anti-collagen type I). Lanes 1 and 10 = Marker; Lanes 2–5 are homogenates of a soleus muscle from one HRHF+TM rat, yet prepared in different manners. Lanes 6–9 is a homogenate of a flexor digitorum muscle from one FRC rat, yet prepared in different manners. Samples in lanes 2 and 6 were boiled and exposed to BME. Samples in lanes 4 and 7 were not boiled before exposure to BME. Samples in lanes 5 and 9 were neither boiled nor exposed to BME. All samples were run on a 4–12% Tris-Glycine gel without SDS in the gel, yet with SDS in the sample and loading buffers. The right image is the same membrane stained with Ponceau S prior to antibody probing, used as a loading control.

## Data Availability

The datasets used and/or analyzed during the current study available from the corresponding author on reasonable request.

## Abbreviations

CD68: Cluster of differentiation 68, a protein highly expressed by cells of the monocyte lineage (phagocytic macrophages) in rodents
FRC: Food restricted control rats
Hr: Hour
HRHF: High repetition high force
IL-10: Interleukin 10
IL-1β: Interleukin 1 beta
m/min: meters/minute
min: minutes
MPF: Maximum pulling force
ms: milliseconds
MSDs: Musculoskeletal disorders
NIH: National Institute of Health
NIOSH: National Institute of Occupational Safety and Health
NORA: National Occupational Research Agenda
Pg/ml: picograms/milliliter
ROI: region of interest
TM: Ttreadmill
TNFα: Tumor necrosis factor alpha
Wk: Week

## References

[1] Employer-Reported Workplace Injuries and Illnesses-2017. www.bls.gov/news.release/osh.nr0.htm. Accessed 17 Oct 2019.

[2] Sauter Steve (2014). Beyond Biomechanics.

[3] Yearout R (1997). Beyond biomechanics: Psychosocial aspects of musculoskeletal disorders in office work - Moon,S, Sauter,SL. Int J Ind Ergonom.

[4] Alavi SS, Makarem J, Abbasi M, Rahimi A, Mehrdad R (2016). Association between upper extremity musculoskeletal disorders and mental health status in office workers. Work.

[5] Russell H, Maitre B, Watson D (2016). Work-related Musculoskeletal Disorders and Stress, Anxiety and Depression in Ireland: Evidence from teh WNHS 2002–2013. Whitacker Square, Sir John Rogerson’s Quay.

[6] Zugel M, Maganaris CN, Wilke J, Jurkat-Rott K, Klingler W, Wearing SC, Findley T, Barbe MF, Steinacker JM, Vleeming A (2018). Fascial tissue research in sports medicine: from molecules to tissue adaptation, injury and diagnostics: consensus statement. Br J Sports Med.

[7] Gold JE, Hallman DM, Hellstrom F, Bjorklund M, Crenshaw AG, Mathiassen SE, Barbe MF, Ali S (2017). Systematic review of quantitative imaging biomarkers for neck and shoulder musculoskeletal disorders. BMC Musculoskelet Disord.

[8] Gold JE, Hallman DM, Hellstrom F, Bjorklund M, Crenshaw AG, Djupsjobacka M, Heiden M, Mathiassen SE, Piligian G, Barbe MF (2016). Systematic review of biochemical biomarkers for neck and upper-extremity musculoskeletal disorders. Scand J Work Environ Health.

[9] Fisher PW, Zhao Y, Rico MC, Massicotte VS, Wade CK, Litvin J, Bove GM, Popoff SN, Barbe MF (2015). Increased CCN2, substance P and tissue fibrosis are associated with sensorimotor declines in a rat model of repetitive overuse injury. J Cell Commun Signal.

[10] Gao HG, Fisher PW, Lambi AG, Wade CK, Barr-Gillespie AE, Popoff SN, Barbe MF (2013). Increased serum and musculotendinous fibrogenic proteins following persistent low-grade inflammation in a rat model of long-term upper extremity overuse. PLoS One.

[11] Barbe MF, Barr AE (2006). Inflammation and the pathophysiology of work-related musculoskeletal disorders. Brain Behav Immun.

[12] Xin DL, Hadrevi J, Elliott ME, Amin M, Harris MY, Barr-Gillespie AE, Barbe MF (2017). Effectiveness of conservative interventions for sickness and pain behaviors induced by a high repetition high force upper extremity task. BMC Neurosci.

[13] Gallo J, Raska M, Kriegova E, Goodman SB (2017). Inflammation and its resolution and the musculoskeletal system. J Orthop Translat.

[14] Yang W, Hu P (2018). Skeletal muscle regeneration is modulated by inflammation. J Orthop Translat.

[15] Zhang JM, An J (2007). Cytokines, inflammation, and pain. Int Anesthesiol Clin.

[16] Handschin C, Spiegelman BM (2008). The role of exercise and PGC1alpha in inflammation and chronic disease. Nature.

[17] Suzuki K. Characterization of Exercise-Induced Cytokine Release, the Impacts on the Body, the Mechanisms and Modulations. Int J Sports Exerc Med. 2019;5:122. 10.23937/2469-5718/1510122.

[18] Pedersen M, Bruunsgaard H, Weis N, Hendel HW, Andreassen BU, Eldrup E, Dela F, Pedersen BK (2003). Circulating levels of TNF-alpha and IL-6-relation to truncal fat mass and muscle mass in healthy elderly individuals and in patients with type-2 diabetes. Mech Ageing Dev.

[19] Arango Duque G, Descoteaux A (2014). Macrophage cytokines: involvement in immunity and infectious diseases. Front Immunol.

[20] Carp SJ, Barbe MF, Winter KA, Amin M, Barr AE (2007). Inflammatory biomarkers increase with severity of upper-extremity overuse disorders. Clin Sci (Lond).

[21] Carp SJ, Barr AE, Barbe MF (2008). Serum biomarkers as signals for risk and severity of work-related musculoskeletal injury. Biomark Med.

[22] Kuiper JI, Verbeek JH, Everts V, Straub JP, Frings-Dresen MH (2005). Serum markers of collagen metabolism: construction workers compared to sedentary workers. Occup Environ Med.

[23] Kuiper JI, Verbeek JH, Straub JP, Everts V, Frings-Dresen MH (2002). Physical workload of student nurses and serum markers of collagen metabolism. Scand J Work Environ Health.

[24] Matute Wilander A, Karedal M, Axmon A, Nordander C (2014). Inflammatory biomarkers in serum in subjects with and without work related neck/shoulder complaints. BMC Musculoskelet Disord.

[25] Pinski SE, King KB, Davidson BS, Zhou BH, Lu Y, Solomonow M (2010). High-frequency loading of lumbar ligaments increases proinflammatory cytokines expression in a feline model of repetitive musculoskeletal disorder. Spine J.

[26] Gold JE, Mohamed FB, Ali S, Barbe MF (2014). Serum and MRI biomarkers in mobile device texting: a pilot study. Hum Factors.

[27] Rechardt M, Shiri R, Matikainen S, Viikari-Juntura E, Karppinen J, Alenius H (2011). Soluble IL-1RII and IL-18 are associated with incipient upper extremity soft tissue disorders. Cytokine.

[28] Smith BE, Hendrick P, Bateman M, Holden S, Littlewood C, Logan P, Smith TO (2019). Musculoskeletal pain and exercise-challenging existing paradigms and introducing new. Br J Sports Med.

[29] Rodrigues EV, Gomes AR, Tanhoffer AI, Leite N (2014). Effects of exercise on pain of musculoskeletal disorders: a systematic review. Acta Ortop Bras.

[30] Kobayashi I, Tanaka A, Okuzumi H (1996). Computer use in schools for the blind in Japan. Psychol Rep.

[31] Suzuki Katsuhiko (2019). Chronic Inflammation as an Immunological Abnormality and Effectiveness of Exercise. Biomolecules.

[32] Starkie R, Ostrowski SR, Jauffred S, Febbraio M, Pedersen BK (2003). Exercise and IL-6 infusion inhibit endotoxin-induced TNF-alpha production in humans. FASEB J.

[33] Gielen S, Adams V, Mobius-Winkler S, Linke A, Erbs S, Yu J, Kempf W, Schubert A, Schuler G, Hambrecht R (2003). Anti-inflammatory effects of exercise training in the skeletal muscle of patients with chronic heart failure. J Am Coll Cardiol.

[34] Brooks SV, Vasilaki A, Larkin LM, McArdle A, Jackson MJ (2008). Repeated bouts of aerobic exercise lead to reductions in skeletal muscle free radical generation and nuclear factor kappaB activation. J Physiol.

[35] Brandt C, Pedersen BK (2010). The role of exercise-induced myokines in muscle homeostasis and the defense against chronic diseases. J Biomed Biotechnol.

[36] Makki K, Froguel P, Wolowczuk I (2013). Adipose tissue in obesity-related inflammation and insulin resistance: cells, cytokines, and chemokines. ISRN Inflamm.

[37] Barbe MF, Gallagher S, Massicotte VS, Tytell M, Popoff SN, Barr-Gillespie AE (2013). The interaction of force and repetition on musculoskeletal and neural tissue responses and sensorimotor behavior in a rat model of work-related musculoskeletal disorders. BMC Musculoskelet Disord.

[38] Clark BD, Al-Shatti TA, Barr AE, Amin M, Barbe MF (2004). Performance of a high-repetition, high-force task induces carpal tunnel syndrome in rats. J Orthop Sports Phys Ther.

[39] Barbe Mary F., Hilliard Brendan A., Delany Sean P., Iannarone Victoria J., Harris Michele Y., Amin Mamta, Cruz Geneva E., Barreto‐Cruz Yeidaliz, Tran Ngih,  Day Emily P., Hobson Lucas J., Assari Soroush, Popoff Steven N. (2019). Blocking CCN2 Reduces Progression of Sensorimotor Declines and Fibrosis in a Rat Model of Chronic Repetitive Overuse. Journal of Orthopaedic Research.

[40] Bove GM, Harris MY, Zhao H, Barbe MF (2016). Manual therapy as an effective treatment for fibrosis in a rat model of upper extremity overuse injury. J Neurol Sci.

[41] Abdelmagid SM, Barr AE, Rico M, Amin M, Litvin J, Popoff SN, Safadi FF, Barbe MF (2012). Performance of repetitive tasks induces decreased grip strength and increased fibrogenic proteins in skeletal muscle: role of force and inflammation. PLoS One.

[42] Kietrys DM, Barr AE, Barbe MF (2011). Exposure to repetitive tasks induces motor changes related to skill acquisition and inflammation in rats. J Mot Behav.

[43] Cote JN (2012). A critical review on physical factors and functional characteristics that may explain a sex/gender difference in work-related neck/shoulder disorders. Ergonomics.

[44] Gender, Health and Work. http://www.who.int/occupational_health/topics/gender/en/. Accessed 17 Oct 2019.

[45] Gender, equity and human rights: Women on the move: Migration, care work and health. http://www.who.int/gender-equity-rights/en/. Accessed 17 Oct 2019.

[46] Barbe MF, Jain NX, Massicotte VS, Popoff SN, Barr-Gillespie AE (2015). Ergonomic task reduction prevents bone osteopenia in a rat model of upper extremity overuse. Ind Health.

[47] Jain NX, Barr-Gillespie AE, Clark BD, Kietrys DM, Wade CK, Litvin J, Popoff SN, Barbe MF (2014). Bone loss from high repetitive high force loading is prevented by ibuprofen treatment. J Musculoskelet Neuronal Interact.

[48] Rani S, Barbe MF, Barr AE, Litivn J (2010). Role of TNF alpha and PLF in bone remodeling in a rat model of repetitive reaching and grasping. J Cell Physiol.

[49] Barbe MF, Massicotte VS, Assari S, Monroy MA, Frara N, Harris MY, Amin M, King T, Cruz GE, Popoff SN (2018). Prolonged high force high repetition pulling induces osteocyte apoptosis and trabecular bone loss in distal radius, while low force high repetition pulling induces bone anabolism. Bone.

[50] Pedersen BK, Steensberg A, Fischer C, Keller C, Ostrowski K, Schjerling P (2001). Exercise and cytokines with particular focus on muscle-derived IL-6. Exerc Immunol Rev.

[51] Peake J, Nosaka K, Suzuki K (2005). Characterization of inflammatory responses to eccentric exercise in humans. Exerc Immunol Rev.

[52] Iannarone VJ, Cruz GE, Hilliard BA, Barbe MF (2019). The answer depends on the question: optimal conditions for western blot characterization of muscle collagen type 1 depends on desired isoform. J Biol Methods.

[53] Fedorczyk JM, Barr AE, Rani S, Gao HG, Amin M, Amin S, Litvin J, Barbe MF (2010). Exposure-dependent increases in IL-1beta, substance P, CTGF, and tendinosis in flexor digitorum tendons with upper extremity repetitive strain injury. J Orthop Res.

[54] Okada K, Arai S, Itoh H, Adachi S, Hayashida M, Nakase H, Ikemoto M (2016). CD68 on rat macrophages binds tightly to S100A8 and S100A9 and helps to regulate the cells' immune functions. J Leukoc Biol.

[55] Brochhausen C, Schmitt VH, Mamilos A, Schmitt C, Planck CN, Rajab TK, Hierlemann H, Kirkpatrick CJ (2017). Expression of CD68 positive macrophages in the use of different barrier materials to prevent peritoneal adhesions-an animal study. J Mater Sci Mater Med.

[56] Rubio-Navarro A, Guerrero-Hue M, Martin-Fernandez B, Cortegano I, Olivares-Alvaro E, de Las HN, Alia M, de Andres B, Gaspar ML, Egido J, et al. Phenotypic characterization of macrophages from rat kidney by flow Cytometry. J Vis Exp. 2016;(116). 10.3791/54599. 10.3791/54599 PMC5092211 27805599

[57] Clark BD, Barr AE, Safadi FF, Beitman L, Al-Shatti T, Amin M, Gaughan JP, Barbe MF (2003). Median nerve trauma in a rat model of work-related musculoskeletal disorder. J Neurotrauma.

[58] Bove GM, Weissner W, Barbe MF (2009). Long lasting recruitment of immune cells and altered epi-perineurial thickness in focal nerve inflammation induced by complete Freund's adjuvant. J Neuroimmunol.

[59] Kjaer M (2004). Role of extracellular matrix in adaptation of tendon and skeletal muscle to mechanical loading. Physiol Rev.

[60] Abd El-Kader SM, Al-Jiffri OH, Ashmawy EM, Gaowgzeh RA (2016). Treadmill walking exercise modulates bone mineral status and inflammatory cytokines in obese asthmatic patients with long term intake of corticosteroids. Afr Health Sci.

[61] Li FH, Sun L, Zhu M, Li T, Gao HE, Wu DS, Zhu L, Duan R, Liu TC (2018). Beneficial alterations in body composition, physical performance, oxidative stress, inflammatory markers, and adipocytokines induced by long-term high-intensity interval training in an aged rat model. Exp Gerontol.

[62] Mathur N, Pedersen BK (2008). Exercise as a mean to control low-grade systemic inflammation. Mediat Inflamm.

[63] Bove GM, Delany SP, Hobson L, Cruz GE, Harris MY, Amin M, Chapelle SL, Barbe MF (2019). Manual therapy prevents onset of nociceptor activity, sensorimotor dysfunction, and neural fibrosis induced by a volitional repetitive task. Pain.

[64] Perry VH, Brown MC, Gordon S (1987). The macrophage response to central and peripheral nerve injury. A possible role for macrophages in regeneration. J Exp Med.

[65] O'Brien JP, Mackinnon SE, MacLean AR, Hudson AR, Dellon AL, Hunter DA (1987). A model of chronic nerve compression in the rat. Ann Plast Surg.

[66] Mackinnon SE, Dellon AL (1986). Experimental study of chronic nerve compression. Clinical implications. Hand Clin.

[67] Mackinnon SE, Dellon AL, Hudson AR, Hunter DA (1986). Chronic human nerve compression--a histological assessment. Neuropathol Appl Neurobiol.

[68] Mackinnon SE, Dellon AL, Hudson AR, Hunter DA (1984). Chronic nerve compression--an experimental model in the rat. Ann Plast Surg.

[69] Kalynovska N, Diallo M, Palecek J (2019). Losartan treatment attenuates the development of neuropathic thermal hyperalgesia induced by peripheral nerve injury in rats. Life Sci.

[70] Pascarelli EF, Hsu YP (2001). Understanding work-related upper extremity disorders: clinical findings in 485 computer users, musicians, and others. J Occup Rehabil.

[71] Klyne DM, Barbe MF, van den Hoorn W, Hodges PW (2018). ISSLS PRIZE IN CLINICAL SCIENCE 2018: longitudinal analysis of inflammatory, psychological, and sleep-related factors following an acute low back pain episode-the good, the bad, and the ugly. Eur Spine J.

[72] Beavers KM, Brinkley TE, Nicklas BJ (2010). Effect of exercise training on chronic inflammation. Clin Chim Acta.

[73] Al-Shatti T, Barr AE, Safadi FF, Amin M, Barbe MF (2005). Increase in inflammatory cytokines in median nerves in a rat model of repetitive motion injury. J Neuroimmunol.

[74] Barbe MF, Elliott MB, Abdelmagid SM, Amin M, Popoff SN, Safadi FF, Barr AE (2008). Serum and tissue cytokines and chemokines increase with repetitive upper extremity tasks. J Orthop Res.

[75] James G, Millecamps M, Stone LS, Hodges PW (2018). Dysregulation of the inflammatory mediators in the Multifidus muscle after spontaneous intervertebral disc degeneration SPARC-null mice is ameliorated by physical activity. Spine (Phila Pa 1976).

[76] Marcus RL, Addison O, Kidde JP, Dibble LE, Lastayo PC (2010). Skeletal muscle fat infiltration: impact of age, inactivity, and exercise. J Nutr Health Aging.

[77] Fu J, Wang H, Deng L, Li J (2016). Exercise training promotes functional recovery after spinal cord injury. Neural Plast.

[78] Shefer G, Rauner G, Stuelsatz P, Benayahu D, Yablonka-Reuveni Z (2013). Moderate-intensity treadmill running promotes expansion of the satellite cell pool in young and old mice. FEBS J.

[79] Asensio-Pinilla E, Udina E, Jaramillo J, Navarro X (2009). Electrical stimulation combined with exercise increase axonal regeneration after peripheral nerve injury. Exp Neurol.

[80] Lima LV, Abner TSS, Sluka KA (2017). Does exercise increase or decrease pain? Central mechanisms underlying these two phenomena. J Physiol.

[81] Staud R, Robinson ME, Price DD (2005). Isometric exercise has opposite effects on central pain mechanisms in fibromyalgia patients compared to normal controls. Pain.

[82] Sluka KA, Danielson J, Rasmussen L, DaSilva LF (2012). Exercise-induced pain requires NMDA receptor activation in the medullary raphe nuclei. Med Sci Sports Exerc.

[83] Moraska A, Deak T, Spencer RL, Roth D, Fleshner M (2000). Treadmill running produces both positive and negative physiological adaptations in Sprague-Dawley rats. Am J Physiol Regul Integr Comp Physiol.

[84] Girard I, Garland T (2002). Plasma corticosterone response to acute and chronic voluntary exercise in female house mice. J Appl Physiol (1985).

[85] Frank MG, Watkins LR, Maier SF (2013). Stress-induced glucocorticoids as a neuroendocrine alarm signal of danger. Brain Behav Immun.

[86] Xin DL, Harris MY, Wade CK, Amin M, Barr AE, Barbe MF (2011). Aging enhances serum cytokine response but not task-induced grip strength declines in a rat model of work-related musculoskeletal disorders. BMC Musculoskelet Disord.

[87] Fischer CP (2006). Interleukin-6 in acute exercise and training: what is the biological relevance?. Exerc Immunol Rev.

[88] Kim M, Yoo JI, Kim MJ, Na JB, Lee SI, Park KS (2019). Prevalence of upper extremity musculoskeletal diseases and disability among fruit tree farmers in Korea: cross-sectional study. Yonsei Med J.

[89] Sharma R, Singh R (2014). Work-related musculoskeletal disorders, job stressors and gender responses in foundry industry. Int J Occup Saf Ergon.

[90] Bovenzi M, Schust M, Menzel G, Prodi A, Mauro M (2015). Relationships of low back outcomes to internal spinal load: a prospective cohort study of professional drivers. Int Arch Occup Environ Health.

[91] Chen JC, Chan WP, Katz JN, Chang WP, Christiani DC (2004). Occupational and personal factors associated with acquired lumbar spondylolisthesis of urban taxi drivers. Occup Environ Med.

[92] Commons KG, Cholanians AB, Babb JA, Ehlinger DG (2017). The rodent forced swim test measures stress-coping strategy, Not Depression-like Behavior. ACS Chem Neurosci.

