# Supplementary figures and images for: Forced treadmill running reduces systemic inflammation yet worsens upper limb discomfort in a rat model of work-related musculoskeletal disorders
Source: BMC Musculoskelet Disord. 2020 Jan 30;21:57. doi: 10.1186/s12891-020-3085-z (PMC6993343; doi:10.1186/s12891-020-3085-z)

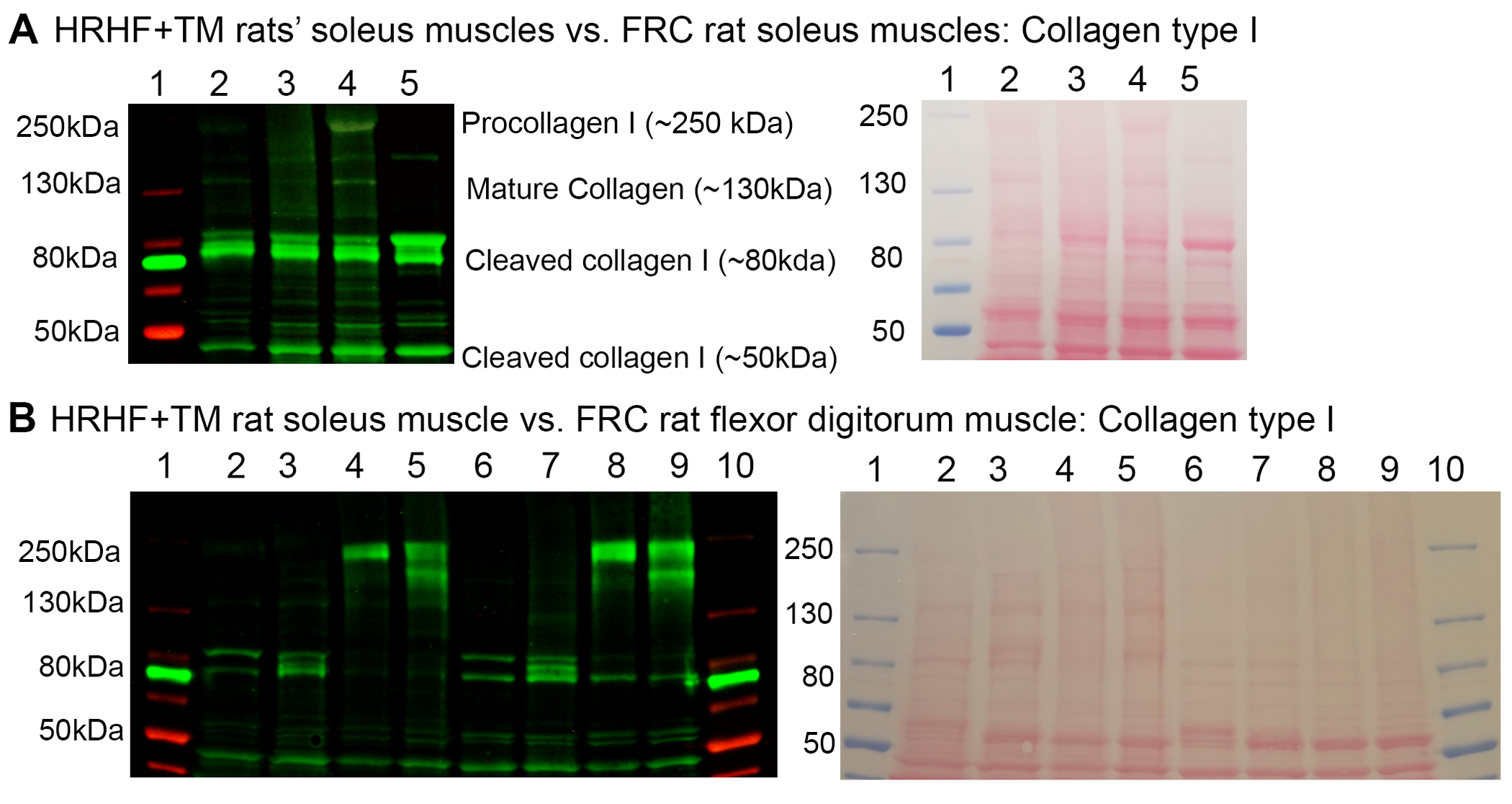

Supplement: Supplementary file 1 — Additional file 1: Figure S1. Western blots of muscles probed for Collagen type I. Expected location of procollagen I, mature collagen type I, and two cleaved collagen bands are indicated. No significant changes in collagen synthesis or cleavage were observed. A) Left panel is a representative Western blot of 10-wk HRHF+TM rats’ soleus muscles and a FRC rat’s soleus muscle, probed with an antibody against collagen type I (Sigma C2456). Lane 1 = Marker; Lanes 2–4 are homogenates of soleus muscles from 3 different 10-wk HRHF+TM rats. Lane 5 is a homogenate of a soleus muscle from a FRC rat. Samples were not boiled, but exposed to BME before running on a 4–12% Tris-Glycine gel without SDS in the gel, yet with SDS in the sample and loading buffers. The right image is the same membrane stained with Ponceau S prior to antibody probing, used as a loading control. B) Left panel is a representative Western blot of a HRHF+TM rat’s soleus muscles compared to a FRC rat’s flexor digitorum muscle, after probing with the same antibody as in panel A (Sigma C2456 anti-collagen type I). Lanes 1 and 10 = Marker; Lanes 2–5 are homogenates of a soleus muscle from one HRHF+TM rat, yet prepared in different manners. Lanes 6–9 is a homogenate of a flexor digitorum muscle from one FRC rat, yet prepared in different manners. Samples in lanes 2 and 6 were boiled and exposed to BME. Samples in lanes 4 and 7 were not boiled before exposure to BME. Samples in lanes 5 and 9 were neither boiled nor exposed to BME. All samples were run on a 4–12% Tris-Glycine gel without SDS in the gel, yet with SDS in the sample and loading buffers. The right image is the same membrane stained with Ponceau S prior to antibody probing, used as a loading control. [file 12891_2020_3085_MOESM1_ESM.tif]
